# Treatment and Prevention of Lung Cancer Using a Virus-Infected Reprogrammed Somatic Cell-Derived Tumor Cell Vaccination (VIReST) Regime

**DOI:** 10.3389/fimmu.2020.01996

**Published:** 2020-08-13

**Authors:** Zhe Zhang, Shuangshuang Lu, Louisa S. Chard Dunmall, Zhizhong Wang, Zhenguo Cheng, Zhongxian Zhang, Wenli Yan, Yongchao Chu, Dongling Gao, Na Wang, Yang Li, Jiwei Wang, Yuenan Li, Yupei Ji, Danyang Shan, Keke Li, Panpan Wang, Yunshu Dong, Jianzeng Dong, Nick R. Lemoine, Duanqing Pei, Lirong Zhang, Yaohe Wang

**Affiliations:** ^1^National Center for International Research in Cell and Gene Therapy, Sino-British Research Centre for Molecular Oncology, School of Basic Medical Sciences, Academy of Medical Sciences, Zhengzhou University, Zhengzhou, China; ^2^National Center for International Research in Cell and Gene Therapy, Sino-British Research Centre for Molecular Oncology, Academy of Medical Sciences, Zhengzhou University, Zhengzhou, China; ^3^Centre for Biomarkers and Biotherapeutics, Barts Cancer Institute, Queen Mary University of London, London, United Kingdom; ^4^CAS Key Laboratory of Infection and Immunity, Institute of Biophysics, Chinese Academy of Sciences, Beijing, China; ^5^Department of Cardiology, Beijing Anzhen Hospital, Capital Medical University, Beijing, China; ^6^CAS Key Laboratory of Regenerative Biology, South China Institute for Stem Cell Biology and Regenerative Medicine, Guangzhou Institutes of Biomedicine and Health, Chinese Academy of Sciences, Guangzhou, China; ^7^School of Basic Medical Sciences, Academy of Medical Sciences, Zhengzhou University, Zhengzhou, China

**Keywords:** lung cancer, vaccine, induced pluripotent stem cells, KRAS, TP53, adenovirus, Vaccinia virus, neo-antigen

## Abstract

Lung cancer is one of the most commonly diagnosed cancer and despite therapeutic advances, mortality remains high. The long period of clinical latency associated with lung cancer provides an ideal window of opportunity to administer vaccines to at-risk individuals that can prevent tumor progression and initiate long-term anti-tumor immune surveillance. Here we describe a personalized vaccination regime that could be applied for both therapeutic and prophylactic prevention of lung cancer, based on the derivation of lung cancer cells from induced pluripotent stem cells. Stem cells from healthy mice were modified to express Cre-dependent KRAS^G12D^ and Trp53^R172H^ prior to differentiation to lung progenitor cells. Subsequent viral delivery of Cre caused activation of exogenous driver mutations, resulting in transformation and development of lung cancer cells. iPSC-derived lung cancer cells were highly antigenically related to lung cancer cells induced in LSL-KRAS^G12D/+^; Trp53^R172H/+^ transgenic mice and were antigenically unrelated to original pluripotent stem cells or pancreatic cancer cells derived using the same technological platform. For vaccination, induced lung cancer cells were infected with oncolytic Adenovirus or Vaccinia virus, to act as vaccine adjuvants, prior to delivery of vaccines sequentially to a murine inducible transgenic model of lung cancer. Application of this Virus-Infected, Reprogrammed Somatic cell-derived Tumor cell (VIReST) regime primed tumor-specific T cell responses that significantly prolonged survival in both subcutaneous post-vaccine challenge models and induced transgenic models of lung cancer, demonstrating that stem cell-derived prophylactic vaccines may be a feasible intervention for treatment or prevention of lung cancer development in at-risk individuals.

## Introduction

Lung cancer remains one of the most fatal malignant tumors. The 2018 GLOBOCAN report suggested that lung cancer is the most commonly diagnosed cancer (11.6% of total cases) and the leading cause of cancer death (18.4% of total cancer deaths) (1) and despite advances in therapeutic approaches, the 5 year survival rate is 16% for the majority of patients whose tumor is not diagnosed at an early, localized stage (2).

Recent immunotherapeutic approaches for the treatment of lung cancer have investigated therapeutic vaccination as a method of controlling disease progression. Theoretically, vaccination strategies are a promising therapeutic option as they can provoke an anergic immune system to activation against one or more tumor cell antigens, resulting in a long-term immune response against the tumor. The majority of these approaches have their basis in targeting specific tumor associated antigens (TAAs) such as epidermal growth factor receptor (EGFR), over-expressed in over half of lung cancer cases, which has been targeted using Cimavax-EGF, an immunogenic recombinant protein vaccine or Mucin-1 (MUC1), also over-expressed in non-small cell lung cancer using Simuvax, a liposomally delivered MUCI peptide. Despite promising early data, these agents have yet to meet primary endpoints in late phase trials (3, 4). TG4010, an alternative MUCI-based vaccine, delivered using a Vaccinia virus (VV) platform is more promising, and evidence of epitope spreading beyond the primary target epitope has been observed clinically (5). The targeting of multiple epitopes will be crucial for successful vaccination strategies to prevent tumor escape from control, however a phase III study investigating a whole cell allogeneic vaccination approach has also failed to demonstrate improved survival (6). The paucity of success of therapeutic vaccination strategies across all cancers can be ascribed to a suboptimal vaccine design, in which inappropriate antigens are targeted or adjuvants are ineffective at breaking immunological tolerance to tumor antigens, and their application in advanced stage disease in which the tumor microenvironment is highly immunosuppressive and refractory to intervention (7). Targeting early pre-malignant lesions, however, may demonstrate more success. These lesions are heavily infiltrated by both adaptive and innate immune cells, with activated phenotypes, suggesting on-going immune responses. Furthermore, immunosuppressive cells are rare at this stage (7).

Recent data suggests a long period of clinical latency of at least 20 years before clinical detection of lung cancer (8). Analysis of the accrual of mutations during lung cancer evolution suggests that lung adenocarcinomas may be particularly suited for early intervention using prophylactic, as opposed to therapeutic vaccination strategies as accrual of known non-synonymous mutations, particularly driver mutations, occur as early molecular events, and 76% of all mutations were detected in all regions of the tumor (9).

We have recently presented a novel, personalized prophylactic vaccination strategy for the prevention of pancreatic ductal adenocarcinoma (PDAC) development in which induced pluripotent stem cells (iPSCs) were induced to PDAC cells via stable knock-in of inactive KRAS^G12D^ and p53^R172H^ prior to lineage differentiation and transformation (10). Importantly, we found that the derived murine tumor cells displayed high antigenic similarities to PDAC cell lines derived from the related KPC or KC transgenic mouse model, demonstrating that the process we used to generate autologous vaccination material was sufficient to model neo-antigen accrual, based on the genetic and epigenetic profile of autologous stem cells, during the earliest stages of tumorigenesis. When pre-infected with replicating oncolytic Adenovirus (AdV) and Vaccinia virus (VV), to act as potent immune adjuvants, and administered to pre-malignant animals in a prime-boost Virus-Infected Reprogrammed Somatic cell-derived Tumor cell vaccination (VIReST) regime, we were able to significantly delay disease development in a robust transgenic mouse model of disease, extending life-span of prophylactically vaccinated animals by up to 51%. Given the latency associated with lung cancer development, in addition to the early and homogenous accrual of truncal mutations, we reasoned that lung cancer would also be particularly responsive to prophylactic therapy. Here we present a VIReST regime targeting lung cancer, in which iPSCs from healthy animals were modified to express inactivated common driver mutations KRAS^G12D^ and p53^R172H^ (11), prior to differentiation into lung progenitor cells and transformation to tumor cells. The iPSC derived lung cancer cell line was able to mimic the characteristics of the lung cancer cell line established from an early-stage lung cancer model. Pre-infection of these cells with AdV and VV prior to delivery into pre-malignant animals was able to induce potent anti-tumor immune responses that safely delayed tumor development when administered prophylactically using a subcutaneous murine model of lung cancer and when administered prior to disease detection in a complex transgenic model of disease. These data suggest VIReST as an effective alternative to autologous whole cancer cell vaccines, that can be delivered prior to or early in disease development, is antigenically compatible with each patient and can engage immune surveillance mechanisms to detect initiation of lung malignancies.

## Materials and Methods

### Cells and Viruses

Murine embryonic fibroblasts (MEFs) from E13.5 WT mice were cultured using Dulbecco's modified Eagles medium (DMEM) supplemented with 10% fetal bovine serum (FBS). MEF feeder cells were inactivated by mitomycin C (MMC) (10 μg/ml for 2.5 h) (MedChemExpress, HY-13316) treatment. iPSCs were cultured using mES medium (DMEM supplemented with 15% FBS (Gemini, 900–108), leukemia inhibitory factor (LIF) (10 ng/ml) (Thermo Fisher Scientific, PMC9484), β-mercaptoethanol (0.1 mM) (Sigma Aldrich, 60-24-2), 1x non-essential amino acids (NEAA) (Thermo Fisher Scientific, 11140050), 1x Glutamax (Thermo Fisher Scientific, 35050061), Sodium pyruvate (1 mM) (Thermo Fisher Scientific, 11360070).

The LC cell lines KPL 160302S and KPL 160424S were cultured from LSL-KRASG12D/+; Trp53R172H/+ mice that had developed lung cancer after Ad-Cre inhalation. These were maintained in DMEM supplemented with 10% FBS. CV1 (African monkey kidney) cells and JH293 cells were obtained from the American Type Culture Collection (ATCC, VA, USA) and maintained in DMEM containing 10% FBS. KP-LC cells were maintained in DMEM containing 10% FBS. The PDAC cell line TB11381 was cultured from LSL-KRASG12D/+; Trp53R172H/+; Pdx-1-Cre mice that had developed PDAC (12). This was kindly provided by David Tuveson (Cancer Research UK Cambridge Institute, now at Cold Spring Harbor Laboratory) and maintained in DMEM supplemented with 10% FBS. Mouse Ovarian Surface Epithelial Cell Line (MOSEC) was provided by Prof Iain McNeish at Barts Cancer Institute and maintained in DMEM supplemented with 10% FBS. All cell lines were routinely tested for the presence of mycoplasma. Cell lines used or created during this study are listed in Table S1.

The thymidine kinase (TK)-deleted Lister strain Vaccinia virus (VVL15) was described previously (13). VVL15 was further modified by *XhoI/EcoRI*-mediated removal of the LacZ open reading frame (ORF) from the VV TK shuttle vector pSC65 (GenBank: HC193923.1), which was replaced with red fluorescent protein (RFP) derived by *NheI/AflII* digestion of pCMV-dsRED-Express (Clontech). The virus was produced as previously described (14). Ad-Cre non-replicative virus was purchased from Vector Biolabs and propagated in our laboratory. Wild-type Adenovirus serotype 5 (Ad5) was described previously (15).

### Teratoma Test

A total 1 × 10^7^ of LSL-KRAS^G12D/+^; LSL-Trp53^R172H/+^ iPSCs were implanted subcutaneously into the right flank of nude male mice. After 2 weeks and 4 days, with diameter of <1.5 cm, tumors were surgically dissected, formalin-fixed, paraffin-embedded, and stained using hematoxylin and eosin (H&E).

### Induction of LSL-KRAS^G12D/+^; LSL-Trp53^R172H/+^ iPSCs to Lung Cancer Cells

LSL-KRAS^G12D/+^; LSL-Trp53^R172H/+^ iPSCs were cultured on mouse embryonic fibroblasts (MEF) in-mES medium. iPSCs were treated with 0.25% trypsin for 5 min, and the differential adhesion method was used to remove MEF. The basic medium of induction was serum-free differentiation (SFD) media of DMEM/F12 (3:1) (Thermo Fisher Scientific, 11765054) supplemented with N2 (Thermo Fisher Scientific, 17502048), B27 (Thermo Fisher Scientific, 12587010), ascorbic acid (50 μg/ml) (Sigma Aldrich), Glutamax (2 mM) (Thermo Fisher Scientific, 35050061), monothioglycerol (0.4 μM) (Sigma Aldrich, 96-27-5), 0.05% bovine serum albumin (BSA) (Sangon Biotech, 9048-46-8), 1% penicillin-streptomycin. iPSCs were plated onto six-well plates coated by 1% agar to form embryoid bodies in the media of SFD with Y-27632 (10 μM) (R&D Systems, 129830-38-2) and mouse BMP4 (3 ng/ml) (R&D Systems, 5020-BP) for 24 h. Embryoid bodies were induced to endoderm in SFD with Y27632 (10 μM), mouse BMP4 (0.5 ng/ml), mouse bFGF (2.5 ng/ml) (R&D Systems, 3139-FB), mouse Activin A (100 ng/ml) (PeproTech, 120-14) for 72 h on low-adherence plates. Half of the old media was removed, and half new media was added every 36 h. Endodermal cells were then induced to anterior foregut endoderm. Cells were cultured on 0.2% Gelatin-coated, 24-well plates (100,000–150,000 cells/well) in SFD with Dorsomorphin dihydrochloride (1.5 μM) (R&D Systems, 1219168-18-9) and SB431542 (10 μM) (R&D Systems, 301836-41-9) for 24 h, followed by SFD with SB431542 (10 μM) and IWP2 (1 μM) (R&D Systems,686770-61-6) for 24 h. Cells were induced to lung progenitors in the medium of SFD with CHIR99021 (3 μM) (Stemgent,04-0004-10), mouse FGF10 (10 ng/ml) (R&D Systems, 6224-FG-025), mouse FGF7 (10 ng/ml) (R&D Systems, 5028-KG-025), mouse BMP4 (10 ng/ml) (R&D Systems, 5020-BP), and *all-trans* retinoic acid (ATRA) (1 μM) (Sigma, 302-79-4) for 9 days. The differentiation protocol was based on a previous report (16). On the 10th day of lung progenitor differentiation, non-replicative Ad5-Cre was added at 50 PFU/cell to remove the LSL cassette. After continuous passage, lung progenitors transformed into lung cancer cells. The changes in cell morphology at each stage of induced differentiation were photographed using the light microscope.

### Immunofluorescence

At each differentiation stage, cells were fixed with 4% PFA for 15 min at room temperature then washed three times with PBS. The slides of cells were permeabilized with PBS + 0.5% Triton X-100 for 20 min and washed three times with PBS. Slides were blocked with 10% goat serum for 30 min and incubated with the primary antibodies at 4°C overnight (>16 h) diluted in PBS. Following incubation, the slides were rinsed with PBS and incubated with secondary antibodies at 37°C for 1 h. The slides were rinsed with PBS and the cell nucleus was stained with DAPI (Sigma Aldrich). The images were visualized using an Olympus BX41 fluorescence microscope. Dilutions and Catalog numbers for primary antibodies were as follows: Anti-Oct4 antibody (1:200 dilution) (Abcam, ab18976), Anti-Nanog antibody (1:200 dilution) (Abcam, ab80892), Anti-FOXA2 antibody (1:300 dilution) (Abcam, ab108422), Anti-SOX17 antibody (1:50 dilution) (Abcam, ab191699), Human/Mouse/Rat SOX2 antibody (1:200 dilution) (R&D Systems, AF2018), Anti-TTF1 antibody (1:200 dilution) (Abcam, ab76013), Anti-SOX9 antibody (1:200 dilution) (Abcam, ab26414). The secondary antibody: Goat Anti-Rabbit IgG H&L (Alexa Fluor 488) (1:500 dilution) (Abcam, ab150077), Rhodamine (TRITC)-conjugated AffiniPure Bovine Anti-Goat IgG (H+L) (1:100 dilution) (Jackson ImmunoResearch, 805-025-180).

### Quantitative Real-Time PCR

At each differentiation stage, total RNA was extracted using Trizol (Invitrogen) and cDNA was synthesized by HiScript Q Select RT SuperMix for qPCR (+gDNA wiper) kit (Vazyme, R133-01). qPCR was carried out using the ABI STEPONE PLUS system and the AceQTM qPCR SYBR Green Master Mix (Vazyme, Q111-02). The primers used are listed in Table S2.

### Identification of Loxp-Stop-Loxp Cassette Removal After Ad5-Cre Addition

Cells were collected and digested in 500 μl/ tube digestion buffer [distilled water + 50 mM Tris-HCl (PH 8.0) + 100 mM EDTA (PH8.0) + 100 mM NaCl + 1% SDS] + 0.1 mg/ml proteinase K for 30 min at 55°C. Cells were then added 250 μl/ tube saturated NaCl solution and 100 μl chloroform and centrifuged 13,523 g at 4°C for 10 min. The upper supernatant was transferred to another tube and 500 μl isopropanol added prior to centrifugation at 13,523 g for 10 min. The supernatant was discarded and 250 μl 70% alcohol was added for 10 min. The tubes were centrifuged 13,523 g for 5 min. The supernatant was discarded and distilled water was added to dissolve the DNA. DNA concentration was measured using the Nanodrop (Thermo Fisher).

Primers on the both sides of LSL cassette were designed. The bands of activated mutations of KRAS/Trp53 were longer than the bands of wide type with one loxp. The fragments with the complete LSL cassettes cannot be amplified in this PCR system.

Primers for KRAS identification: F: 5′-ATATCCAGTCAACAAAGAATACC-3′, R: 5′-TCCGAATTCAGTGACTACAGATGTACAGAG-3′
Primers for p53 identification: F: 5′-AGCCTGCCTAGCTTCCTCAGG-3′, R: 5′-CTTGGAGACATAGCCACACTG-3′.

### Karyotype Analysis

WT iPSC/KP-LC/KPL 160302S/KPL 160424S were cultured to a density of about 80–90%, and treated with colchicine (0.2 μg/ml) for 3 h. Cells were digested with trypsin and collected into centrifuge tubes, pipetted into single cell suspensions, centrifuged at 250 g for 5 min and the supernatant was discarded. Five milliliters 37°C preheated KCl solution (0.075 mol/L) was added. The cell suspensions were mixed uniformly, and the hypotonic treatment performed in the 37°C water bath for 30 min. The cell suspensions were centrifuged and the supernatant discarded. The cell pellets were resuspended with a little residual liquid. One milliliter fixative solution (methanol: glacial acetic acid = 3: 1) was added slowly along the tube wall. The cell suspensions were mixed while dropping, and left to stand for 5 min. Afterwards, the cell suspensions were centrifuged, and the supernatant was discarded. Three milliliters fixative solution was added, and left to stand for 30 min. Again, the cell suspensions were centrifuged, and the supernatant was discarded. Three milliliters fixative solution was added, and left to stand for 30 min. The cell suspensions were centrifuged, and the supernatant was discarded. One milliliter fixative solution was added, and the cells were pipetted to make cell suspensions. The cell suspension was aspirated with a pipette and dropped on cold glass slides at high altitude. The slides were baked in an oven at 80°C for 2 h, digested by preheating trypsin for 30 s, stained by Giemsa for 10 min, rinsed with water, and dried. Finally, the slides were observed and pictured with oil lens.

### Transcriptome Sequencing

Cell transcriptomes were sequenced in BGI. Tec, Shenzhen, China. RNA libraries were prepared using an Illumina TruSeq RNA Sample Prep Kit. Briefly, 200 ng total RNA sample was purified by oligo-dT beads, then poly (A)-containing mRNA were fragmented into small pieces with Elute, Prime, Fragment mix. First-strand cDNA was generated by First Strand Master Mix and Super Script II (Invitrogen) reverse transcription. The Second Strand Master Mix was added to synthesize second-strand cDNA (16°C for 1 h). The purified fragmented cDNA was combined with end-repair mix and incubated at 30°C for 30 min. The end-repaired DNA was purified using Ampure XP Beads (AGENCOURT). A-Tailing Mix was then added and samples were incubated at 37°C for 30 min. These samples were incubated with RNA Index Adapter and Ligation Mix at 30°C for 10 min. The end-repaired DNA was purified using Ampure XP Beads (AGENCOURT). Several rounds of PCR amplification with a PCR Primer Cocktail and PCR Master Mix were performed to enrich the cDNA fragments. The PCR products were finally purified with Ampure XP Beads (AGENCOURT).

After the libraries were obtained, they were amplified on cBot to generate a cluster on the flowcell (TruSeq PE Cluster Kit V3–cBot–HS, Illumina). The amplified flowcell was sequenced using the HiSeq 2000 System (TruSeq SBS KIT-HS V3, Illumina) with a read length of 100 bp, producing on average 24M paired-end sequence reads per sample. Sequencing reads were aligned to the mouse genome build mm10/GRCm38 with the HISAT2 aligner (17). The number of reads uniquely aligned (mapping quality score *q* > 10) to the exonic region of each gene were counted using HTSeq (18), based on GENCODE version 9 mouse gene annotation. Only genes that achieved at least one count per million (CPM) mapped reads in at least one sample were included, leading to 15,687 filtered genes in total. Read counts were further normalized using the conditional quantile normalization (cqn) method (19), accounting for gene length and GC content, with the FPKM (Fragments Per Kilobase of transcript per Million mapped fragments) values derived for genes across the eight samples. The overall gene expression correlations between samples were subsequently calculated. The data of transcriptome sequencing has been uploaded to GEO, the series entry number is GSE151813. The genes expressed at higher levels in KP-LC compared to WT-iPSCs or KP-LC compared to KPL 160424S were analyzed. Tumor-associated antigens such as CTA, CEA were searched among them. Meanwhile, GO analysis was applied to determine highly expressed genes in KP-LC using the https://david.ncifcrf.gov/summary.jsp~website.

### Cytotoxicity Assay

Cells were seeded at 4 × 10^3^ to 6 × 10^3^ cells per well in 96-well plates and infected with Ad5/ VVL15-RFP 14–18 h later at starting multiplicity of infection (MOI) of 1,000 PFU/cell. Cells were incubated at 37°C, 5% CO_2_ incubator for 6 days after viral infection. Cell survival was detected by SpectraMax M5e microplate reader.

### Viral Replication Assay

Cells were seeded at 2 × 10^5^ cells per well in 24-well plates. Fourteen to eighteen hours later, cells from three wells were harvested with trypsin and cell number per well-determined. Cells were infected with Ad5 (MOI:10 PFU/cell) for 4 h or VVL15-RFP (MOI: 1 PFU/cell) for 2 h. Twenty-four wells were then switched to DMEM + 10% FBS. The other 24 wells were switched to DMEM + 10% FBS + 200 μg/ml MMC for 2.5 h, and then DMEM + 10% FBS. Cells were collected in triplicate at 24-h intervals up to 96 h after infection, freeze-thawed three times and titrated on JH293 cells for Ad5 or CV1 cells for VVL15-RFP. The Reed-Muench mathematical method was used to calculate the 50% tissue culture infective dose (TCID50) value for each sample (20). Viral burst titers were converted to PFU per cell based on the number of cells present at viral infection.

### Cell Proliferation and Plate Colony Formation of KP-LC With Ad5/VVL15-RFP Infection and MMC Treatment

Cells were seeded at 3,000 cells per well in 96-well plates. Fourteen to eighteen hours later, cells were infected with Ad5 (MOI: 50 PFU/cell) for 4 h/VVL15-RFP (MOI: 1 PFU/cell) for 2 h. Then the medium was switched to DMEM + 10% FBS + 200 μg/ml MMC for 2.5 h, and then DMEM + 10% FBS. Cell survival 24 or 72 h after MMC treatment was determined by MTS assay by SpectraMax M5e microplate reader.

In six-well plates, 2.5 × 10^5^ cells per well were seeded. After 14–18 h, cells in 3 wells were digested with trypsin and the average number of cells in each well was calculated. Cells in the remaining wells were infected with Ad5 (MOI: 50 PFU/cell) for 4 h or VVL15-RFP (MOI: 1 PFU/cell) for 2 h. After that, the medium was changed to DMEM + 10% FBS + 200 μg/ml MMC and incubated for 2.5 h, and then the medium was changed to DMEM + 10% FBS. Forty-eight and ninety-six hours after MMC treatment, cells were digested with trypsin and the average number of cells per well was calculated. After counting, the cells were seeded in new six-well plates for plate colony formation experiments. Meanwhile, KP-LC without virus infection and MMC treatment was seeded into six-well plates at 300 or 600 cells per well as a normal control. Six days later, cells of the normal control group grew into visible colonies. The six-well plates were fixed with 4% paraformaldehyde at room temperature for 15 min, and washed twice with PBS. The wells were stained with crystal violet for 20 min, rinsed with tap water and photographed.

### Cell Growth Curve

6 × 10^4^ cells/well were cultured in 96-well plates for 5 days in DMEM + 10% FBS. Cell counts were carried out using Incucyte every 2 h.

### Soft Agar Colony Formation Assay

Two percent agar and DMEM + FBS were mixed to a final concentration of 0.5% agar and 20% FBS, and the mixture placed in to 24-well plates as a base layer. Cells were trypsinized and suspended in DMEM + 20% FBS. 0.5% agar and cells suspension were mixed to a final concentration of 0.2% agar and added over the solid base layer. DMEM + 10% FBS was added after top layer was solidified. Plates were incubated at 37°C for 2 weeks. Colonies of each well were visualized using an Olympus IX51 microscope, counted and colony forming efficiency (total number of colonies/initial number of cells) was calculated.

### Plate Formation Assay

50/100/200 cells/well were seeded in 96-well plates in DMEM + 10% FBS. The plates were then put into Incucyte to take pictures. Two weeks later, colonies of each well were counted, averaged and colony forming efficiency calculated as above.

### Wound Healing Assay

3 × 10^4^ cells/well were cultured in 96-well plates for 1 day. Plates were then scratched with the 96 well pin block and put into Incucyte. Pictures were taken every 2 h and relative wound density was calculated.

### Western Blot

Cells were seeded at 1 × 10^6^ cells per well in six-well plates. Fourteen to eighteen hours later the cell number per well was determined. Cells were infected with Ad5 (MOI: 50 PFU/cell) for 4 h/VVL15-RFP (MOI: 1 PFU/cell) for 2 h. The medium was switched to DMEM + 10% FBS + 200 μg/ml MMC for 2.5 h, and then DMEM + 10% FBS. Cells were collected at 24 and 72 h after MMC treatment. Cells were lysed by RIPA lysis buffer + PMSF (Solarbio, R0020), and concentration of protein was detected by BCA kit (Solarbio, PC0020). Protein was separated by SDS-PAGE gel (CWBIO, CW0022S), and transferred to a piece of PVDF membrane (BEIJING DINGGUO CHANGSHENG BIOTECHNOLOGY CO., XLL094-2). The membrane was blocked with 10% skim milk for 1 h and incubated with the primary antibodies at 4°C overnight diluted in 5% skim milk.

Following incubation, the membrane was rinsed with TBST 3 times and incubated with secondary antibodies diluted in 5% skim milk at room temperature for 1 h. The membrane was rinsed 3 times with TBST and ECL added (Thermo Scientific, NC15079) prior to exposure (GE Amersham Imager 600). Dilutions and catalog numbers for primary antibodies were as follows: Adenovirus Type 5 E1A Ab-1, Mouse Monoclonal Antibody (1:300 dilution) (Thermo, MS-587-P1), Vaccinia virus (Polyclonal Antibody) (1:300 dilution) (AbD Serotec, 9503-2057), GAPDH Antibody (Mouse Monoclonal) (1:5,000 dilution) (Proteintech, 60004-1-Ig). The secondary antibody: Peroxidase-Conjugated Goat anti-Mouse IgG (1:5,000 dilution) (ZSGB-BIO, ZB-2305), Peroxidase-Conjugated Goat anti-Rabbit IgG (1:5,000 dilution) (ZSGB-BIO, ZB-2301).

### *In vivo* Experiments

All animal procedures were approved by the Animal Welfare and Research Ethics Committee of Zhengzhou University (Zhengzhou, China). Mice were housed in groups in accordance with the regulations for mouse welfare and ethics of Zhengzhou University with 12 h dark-light cycles and free access to food and water.

LSL-KRAS^G12D/+^; LSL-Trp53^R172H/+^ (KP) mice were kindly provided by David Tuveson. Genotyping was performed using the following primers; KRAS F: 5′-CCATGGCTTGAGTAAGTCTGC-3′ KRAS R 5′-CGCAGACTGTAGAGCAGCG-3′ (550 bp); P53_F: 5′-AGCTAGCCACCATGGCTTGAGTAAGTCTGCA-3′ P53_R: 5′-CTTGGAGACATAGCCACACTG-3′ (270 bp). This model is modified heterozygous KRAS^G12D^ and Trp53^R172H^ on 129 mouse background. These two point mutations were silenced by a loxp-stop-loxp (LSL) cassette in the absence of Cre. 2.5 × 10^7^ PFU Ad-Cre was delivered to 10–12 week (20–28 g) male mice intranasally. After the intranasal inhalation of Ad-Cre, the two mutations were activated and lung cancer developed gradually. To acquire lung cancer cell lines from this model, lungs of KP mice that had been infected with Ad-Cre were surgically dissected at 8 and 16 weeks, and rinsed in PBS twice. Lungs were dissected in a small amount of PBS + 10% BSA and the cells cultured in ACL-4 media (RPMI 1640 with 20 μg/ml insulin, 10 μg/ml transferrin, 25 nM sodium selenite, 50 nM hydrocortisone, 1 ng/ml epidermal growth factor, 10 μM ethanolamine, 10 μM phosphorylethanolamine, 100 pM triiodothyronine, 2 mg/ml bovine serum albumin, 0.5 mM HEPES, 0.5 mM sodium pyruvate and 2 mM glutamine). After 2 passages, ACL-4 media was switched to DMEM + 10% FBS.

Male C57/BL6 mice and nude mice were purchased from Vitalriver.com, Beijing, China.

In subcutaneous tumor models, 4 to 5-weeks old, 16–18 g male mice were randomly assigned to treatment groups and tumor growth was measured using electronic calipers [tumor volume = (length × width^2^ × π)/6] until tumor volume reached 2,500 mm^3^ or ulcerated, at which point the animal was sacrificed. For KP lung cancer models, animals were assigned randomly to treatment groups and animal survival was monitored by assessment of animal well-being every other day. In the survival experiments, the animal was sacrificed when one of the following situations occurred: animal was curled up motionlessly, hair became fluffy and messy, animal showed no response to external stimuli, weight loss exceeding 2 g in 2 days. Lung tissue was collected from each mouse to confirm, using H&E staining, that all mice developed lung cancer. Animal caretakers were blinded to treatment groups in all cases.

Statistical analysis was carried out using Graph Pad Prism 5 and SPSS 19.0 software. The results were represented as mean ± standard or deviation (SD) or ± standard error of the mean (SEM). Differences between groups were analyzed using Students' unpaired *T*-tests or Kaplan–Meier survival analysis. Differences were considered statistically significant when the *p* < 0.05.

### VIReST Vaccinations for Subcutaneous Tumor Experiments

KP-LC cells were infected with Ad5 at an MOI of 50 PFU/cell in serum-free DMEM for 4 h. Following infection, medium was switched to DMEM + 10% FBS + 200 μg/ml mitomycin C (MMC) (Meilun Biotechnology, 50-07-7) and cells incubated at 37°C for 2.5 h. Cells were washed with PBS twice, harvested with trypsin and diluted with PBS to 2 × 10^6^ cells/100 μl. Ad5-infected cells were injected subcutaneously (s.c) as a prime vaccination using 100 μl per inoculation in the right flank of male KP littermates. KP-LC cells were infected with VVL15 at an MOI of 1 PFU/cell for 2 h and then MMC-treated as for AdV-infected cells. Four weeks after prime vaccination, cells treated with VVL15-MMC were injected s.c in the same side as a booster vaccination. Two weeks after boost, KPL160302S/KPL 160424S cells were injected s.c in 100 μl (2 × 10^6^ cells) per inoculation, in the right flank and tumor growth was monitored.

### Immunohistochemistry

The tissues were collected at different time points, dipped in isopentane and frozen at −80°C. Tissues were cut into 6 μm sections by freezing microtome (Leica, CM1950). The sections were fixed with −20°C acetone for 10 min, washed three times with PBS. Then the slides were incubated with 3% H_2_O_2_ for 8 min, washed twice with PBS and slides blocked with 10% goat serum for 30 min. Slides were incubated with the primary antibodies at 4°C overnight diluted in PBS. Following incubation, the slides were rinsed 3 times with PBS. Polink-2 plus polymer HRP detection system for rat primary antibody (ZSGB-BIO, PV-9004) and DAB kit (ZSGB-BIO, ZLI-9018) were used. The slides were flushed using tap water thoroughly, dyed by hematoxylin, flushed by tap water, differentiated by hydrochloric acid alcohol, dehydrated, and sealed. The images were visualized using an Olympus BX41 microscope. Dilutions and catalog numbers for primary antibodies were as follows: CD4 antibody (1:100 dilution) (BioLegend, 100402), CD8 antibody (1:100 dilution) (BioLegend, 100702).

Ten high-power fields (HPF) were randomly selected from each group to count lymphocytes, three mice per group at each time point. Statistical graphs show the mean ± standard error of the mean (SEM) of each group and compared using independent *T*-test.

### Establishment of Orthotopic Lung Cancer Model

Ad-Cre was intranasally delivered to KRAS^LSL−G12D/+^; p53^LSL−R172H/+^ (KP) mice between 10 and 12 weeks of age. DMEM + Ad5-Cre + 10 mM CaCl_2_ were incubated at room temperature for 20 min to form calcium phosphate precipitates. Meanwhile, mice were anesthetized using avertin at 0.45 mg/g body weight for males via intra-peritoneal injection. A total volume of 75 μl containing 2.5 × 10^7^ PFU per mouse was delivered as previously described (21).

### Vaccinations for Survival Experiments

Ad5-infected KP-LC, KPL 160302S, or KPL 160424S cells produced as described above were injected s.c, using 100 μl per inoculation, in the right flank of male KP mice 2 weeks 4 days after Ad-Cre inhalation, as a prime. Four weeks later, cells treated with VVL15-MMC were injected subcutaneously in the same side as a boost. For analysis of α-PD1 combination, 600 μg α-PD1 (Bioxcell) was administered to mice intra-peritoneally (i.p.) 2 weeks post-prime and 1 and 3 weeks post-booster injection.

### Flow Cytometric Analysis

Splenocytes of three mice vaccinated by KP-LC and 3 mice vaccinated by PBS were stained with CD3e (FITC) (eBioscience, 11-0031-86), CD4 (APC) (eBioscience, 17-0041-82), CD8a (PE) (eBioscience, 12-0081-85), CD44 (eFluor 450) (eBioscience, 48-0441-80), CD62L (PerCP-Cyanine5.5) (eBioscience, 4300748) for 30 min at 4°C. After washing, stained cells were analyzed on an ACEA NovoCyte flow cytometer.

### IFNγ Expression Induced by Vaccination

Spleens of three mice vaccinated using KP-LC and three mice vaccinated using PBS 3 weeks after boost were harvested under sterile conditions. Spleens were mashed through 70 μm cell strainers (BIOFIL, CSS010070), centrifuged at and re-suspended in 5 ml of RBC lysis buffer, washed with PBS, centrifuged and re-suspended with T cell media (TCM) (RPMI-1640 + 10% FBS + 1% streptomycin/ penicillin + 1% sodium pyruvate + 1% non-essential amino acids) to a final concentration of 5 × 10^6^ cells/ml.

KP-LC, KPL 160302S, KPL160424S, KPL-234S, and MOSEC as stimulator cells were incubated with TCM + 0.2 mg/ml MMC for 2.5 h. The cells were washed twice with PBS, trypsinized and re-suspended in TCM to a final concentration of 5 × 10^5^ cells/ml. Peptides of K-RAS (GADGVGKSA) (GL Biochem, 492748), B8R (TSYKFESV) (GL Biochem, 492745), OVA(SIINFEKL) (GL Biochem, 492746) were diluted in TCM to 100 μg/ml.

One hundred microliters of each of the splenocyte suspensions were co-cultured with 100 μl of stimulator cell suspension or peptide in a round bottomed 96 well plate. Background contained 5 × 10^5^ splenocytes in 200 μl TCM. The plates were incubated at 37°C, 5% CO_2_ for 3 days. The plates were centrifuged at 512 g for 5 min. The concentration of IFNγ in supernatants was measured using mouse IFNγ ELISA Ready-SET-Go kit (eBioscience, 88-7314-88).

### *In vivo* CD8+ or CD4+ T Cell Depletion

α-CD8 (TIB2100) or α-CD4 (GK1.5) (Provided by Professor Shengdian Wang, the Chinese Academy of Sciences, Institute of Biophysics) was injected into the abdominal cavity of the KP mice 1 day before prime and two times/week in the interval between prime and boost, and 4 weeks after boost at 200 μg/mouse/time point. CD8+ or CD4+ T cell depletion was confirmed using flow cytometric analysis throughout the experiment.

### Mouse Anti-nuclear Antibody ELISA

Three mice vaccinated using KP-LC and three mice vaccinated using PBS had blood collected from tail tips 3 weeks after boost. The blood was coagulated at 37°C for 15 min and centrifuged 20 min at 845 g. Supernatant was serum, and the concentration of anti-nuclear antibody in serum was measured using mouse anti-nuclear antibody kit (Nanjing Senbeijia Biological Technology Co., SBJ-MO134-96T).

### Statistical Analysis

GraphPad Prism 6 was used for comparative statistical analysis. Dual condition comparisons were made using the unpaired student *T*-test. For more than one condition or for an additional variable such as time, one or two-way ANOVAs, respectively were performed, with *post-hoc* Tukey tests to compare treatment pairs. Survival data was represented by Kaplan-Meier plots with log rank analyses to delineate whether any differences between specific treatment pairs were statistically significant. ^*^p < 0.05, ^**^p < 0.01, and ^***^p < 0.001.

## Results

### Lineage Differentiation and Transformation of iPSCs Provides an Antigenically Compatible Whole Cell Lung Cancer Vaccine

**Figure 1:**
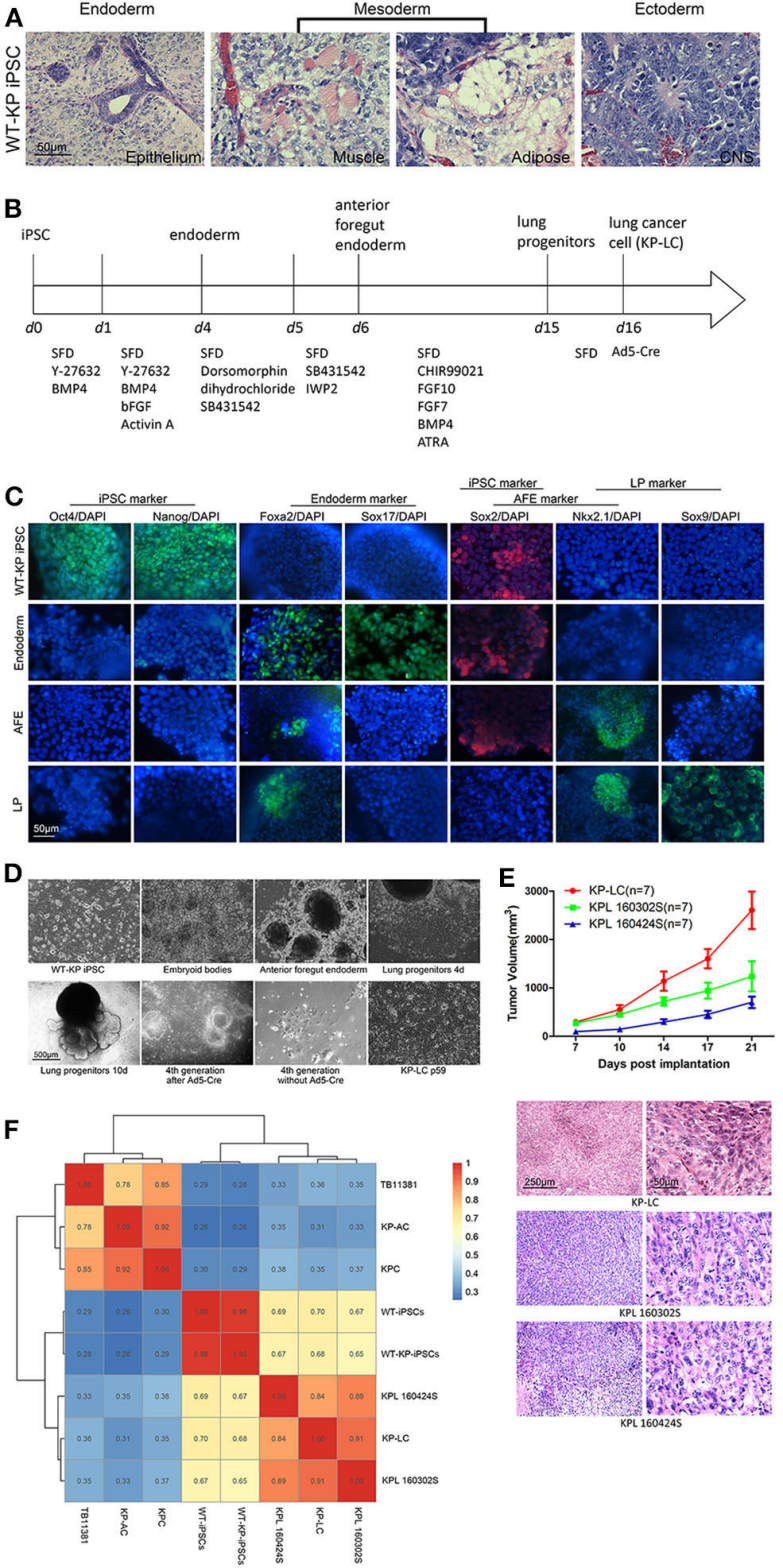
Antigenically relevant lung cancer cells can be derived from murine iPSCs. **(A)** H&E staining of tissues derived after LSL-KRAS^G12D/+^; LSL-Trp53^R172H/+^ iPSCs were subcutaneously injected into nude mice to form teratoma. **(B)** Schematic of the protocol employed for stepwise differentiation of LSL-KRAS^G12D/+^; LSL-Trp53^R172H/+^ iPSCs to lung cancer cells. **(C)** Immunofluorescence images to examine expression of markers indicative of stages of induction of lung progenitor cells from iPSC. DAPI was used to stain for nuclear content. **(D)** Morphology of WT-KP iPSCs, embryoid bodies, anterior foregut endoderm, lung progenitors, and KP-LC. On the 10th day of lung progenitors, cells displayed structures akin to lung buds. Tubular structures could be observed at the 4th generation after infection of Ad5-Cre. Without the infection of Ad5-Cre, lung progenitors gradually became senescent. After serial passage post-infection of Ad5-Cre, cells transformed to lung cancer cells. **(E)** Subcutaneous tumor growth curve of KP littermates bearing KP-LC, KPL 160302S, and KPL 160424S tumors with the initial dose of 2 × 10^6^ cells/ mouse. Mean ± SEM is shown. *N* = 7/group. H&E staining of subcutaneous tumors at day 21 is shown beneath the growth curve. **(F)** Indicated cell lines were subjected to RNA deep sequencing and a transcriptome expression correlation matrix, based on 15,687 filtered genes, was generated.

We have previously generated a wide type iPS cell line from 129J/C57/BL6 male mice and modified the genome with Cre-dependent LSL-KRASG12D/+; LSL-Trp53R172H/+, to create WT-KP iPSCs. The introduction of driver mutations prior to lineage differentiation had no impact on the pluripotency of iPSCs as demonstrated by the ability to form characteristic teratoma when inoculated into nude mice (Figure 1A). WT-KP iPSCs were directed to differentiation to lung progenitor cells following the protocol shown in Figure 1B. To confirm effective induction, markers of iPSC, endoderm, anterior foregut endoderm (AFE), and lung progenitors (LP) were detected by immunofluorescence (Figure 1C) and RT-qPCR (Figure S1A). iPSC-derived LP cells were then infected with non-replicating Ad5 vector expressing Cre (AdCre) to induce mutant KRAS^G12D^ and p53^R172H^ expression and transformation to lung cancer cells (KP-LC) (Figure 1D). Genotyping confirmed the presence of mutant KRAS in the KP-LC cell line and KPL 160302S and KPL160424S cell lines [lung adenocarcinoma cell lines derived from the lung cancer KP transgenic mouse model (21)], but not in LP cells that were not infected with AdCre. Similarly, mutant p53 was detected in KP-LC cell line but not in the AdCre uninfected lung progenitor (LP) cells that do not express mutated p53 (Figure S1B). Furthermore, KP-LC and KPL 160302S, KPL 160424S all had abnormal chromosome profiles (Figure S1C). These three lung cancer cell lines had similar growth rate *in vitro* (Figure S1D). Investigations into the tumorigenicity of iPSC-derived KP-LC cells demonstrated these cells were able to develop into subcutaneous tumors when inoculated into the flank of KP transgenic littermate mice or C57/BL6 mice that had no pre-disposition to cancer (Figure 1E and Figure S1E), forming tumors pathologically similar to those formed after inoculation of KPL 160302S and KPL 160424S tumor cells derived from the induced KP transgenic lung cancer model (Figure 1E and Figure S1E). Scratch assays to examine *in vitro* invasion of cells demonstrated that over 90% of the wound of KPL 160424S cells, from an advanced lung cancer model, healed 16 h after scratch. At the same time point, the wound in KPL 160302S cells, which came from an early stage lung cancer model, demonstrated healing at <20% and KP-LC cells showed a 40% heal from scratch (Figure S1F). Both KP-LC and KPL 160302S were able to form colonies in the soft agar (Figure S1G) and plate colony formation assay (Figure S1H). Most importantly, analysis of the transcriptome of KP-LC cells demonstrated a high similarity between KP-LC cells derived from iPSCs and the cell lines derived from the transgenic mouse model (KPL 160302S and KPL 160424S) (Figure 1F). KP-LC cells had the highest similarity with KPL 160302S which came from the early stage lung cancer model (91%), followed by KPL 160424S which came from the advanced lung cancer model (84%). We speculate that KP-LC derived from iPSC retained more neoantigens/tumor-associated antigens similar to cells from early staged lung cancer model because it had not been screened by the immune system. Similarity between KP-LC and untransformed iPSCs (WT iPSC derived from KPC transgenic mice with wild-type KRAS and p53 or WT-KP iPSCs derived from littermate mice with stable insertion of KRAS^G12D^ and p53^R172H^) was lower, demonstrating the limitations of vaccination strategies based on the use of unmodified iPSCs as previously reported (22). Cancer associated genes such as MageE1, Morc2b, Morc4, Cage1, Brdt, IL13ra1, were expressed at significantly higher levels in KP-LC than WT-iPSCs (Figure S2A) and these genes were enriched in several pathways associated with cancer, including the PI3K-Akt signaling pathway, cancer proteoglycans, the Ras signaling pathway, cancer associated MicroRNAs, the Rap1 signaling pathway, HTLV-1 infection, the MAPK signaling pathway and ECM-receptor interactions (Figure S2C). Furthermore, when compared to KPC or KP-AC iPSCs, which were derived in the same way, using the same driver mutations, but induced to pancreatic lineage tumor cells, and TB11381 cells derived from the transgenic pancreatic cancer model with the same driver mutations, concordance was low. This demonstrates that the lineage specific genetic and epigenetic profiles and not the driver mutation *per se* drive the accumulation of neo-antigens or/and tumor-associated antigens (Figure 1F).

### Oncolytic Viruses Can Successfully Infect and Express Viral Proteins in KP-LC Cells

**Figure 2:**
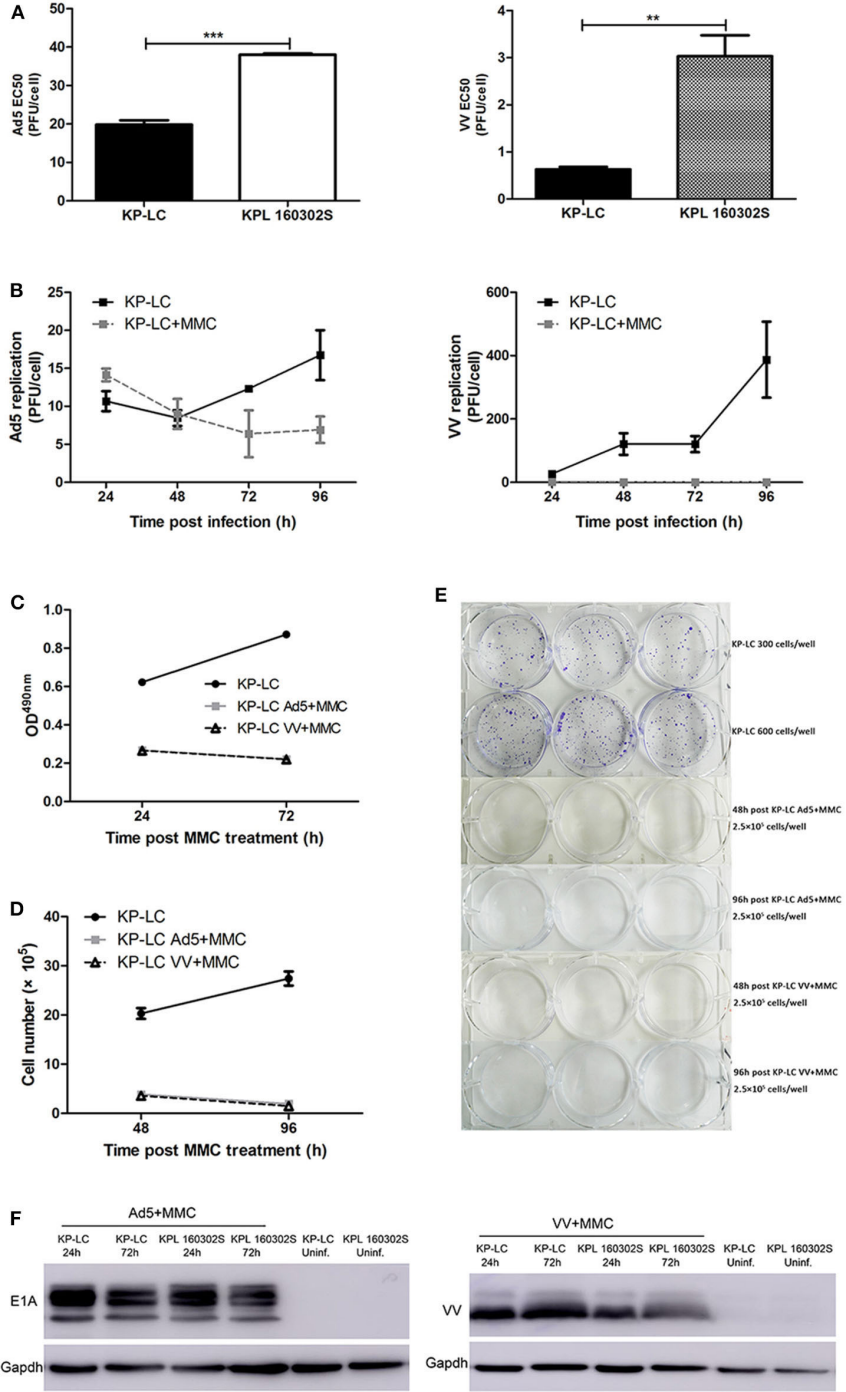
AdV and VV can infect and replicate in transformed iPSCs and mitomycin-C treatment inhibits ongoing replication and tumor cell proliferation. **(A)** Cytotoxicity of Ad5 or VV on iPSC-derived KP-LC cells and KPL 160302S lung tumor cells. Cell death was determined by MTS assay 144 h post-infection. Mean EC50 values ± SEM are shown. **(B)** Production of infectious Ad5 or VV virions in KP-LC cells. Cells were infected with virus and were untreated or treated with mitomycin C. Mean viral replication ± SEM was determined at 24 h intervals for 96 h by TCID50 assay. JH293 cells for Ad5 or CV1 cells for VVL15-RFP. **(C)** Cell proliferation of KP-LC cells after infection and mitomycin-C treatment was determined using MTS assay at 24 and 72 h post-mitomycin C treatment. Mean OD^490^nm values ± SEM are shown. *n* = 3/group. **(D)** Cell proliferation of KP-LC cells after infection and mitomycin-C treatment was determined by cell counting at 48 and 96 h post-mitomycin C treatment. *n* = 3/group. **(E)** Plate colony formation of KP-LC cells after infection and mitomycin-C treatment. **(F)** Viral protein expression was determined in KP-LC or KPL 160302S cells at 24 and 72 h post-infection +/– mitomycin C treatment of cells. Anti-E1A was used to confirm AdV protein expression. Anti-VV coat protein was used to confirm VV protein expression. GAPDH was used as a loading control. **p < 0.01 and ***p < 0.001.

Lack of immunogenicity plays a central role in therapeutic tumor vaccine failure, thus provision of an effective adjuvant is critical for inducing robust anti-tumor immune activity, even within the pre-malignant environment. Virus-infected cell vaccines, whereby tumor cells are pre-infected with replicating tumor tropic virus prior to delivery as a vaccine, has previously been shown to induce strong anti-tumor immune reactions (23), and we have demonstrated that replicating oncolytic Vaccinia virus (VV) and Adenovirus (AdV) can induce the necessary danger signals and inflammatory environment required for anti-tumor immune induction (10). To validate their use in a lung cancer vaccination regime, we investigated the ability of VV and AdV to replicate in and kill iPSC-derived KP-LC cells *in vitro*. Both viruses were significantly more cytotoxic in KP-LC cells compared to KPL160302S cells derived from the induced transgenic mouse model (Figure 2A). Consistent with previous reports (15), Ad5 replicated poorly in murine cells, including KP-LC (Figure 2B), but viral protein expression was detected after infection (Figure 2F). VV replicated and expressed viral protein efficiently in KP-LC cells (Figures 2B,F). In order to ensure safety of the vaccination protocol, we completely inhibited ongoing cell proliferation and virus replication using mitomycin-C (MMC) (Figures 2B–E). This had the effect of preventing both cell proliferation and viral replication, but viral protein expression, required for adequate stimulation of anti-tumor immune responses, persisted for at least 72 h post-infection (Figure 2F).

### A VIReST Regime Using OV-Infected iPSC-Derived KP-LC Cells Prevents Tumor Growth in Immunocompetent Mice

**Figure 3:**
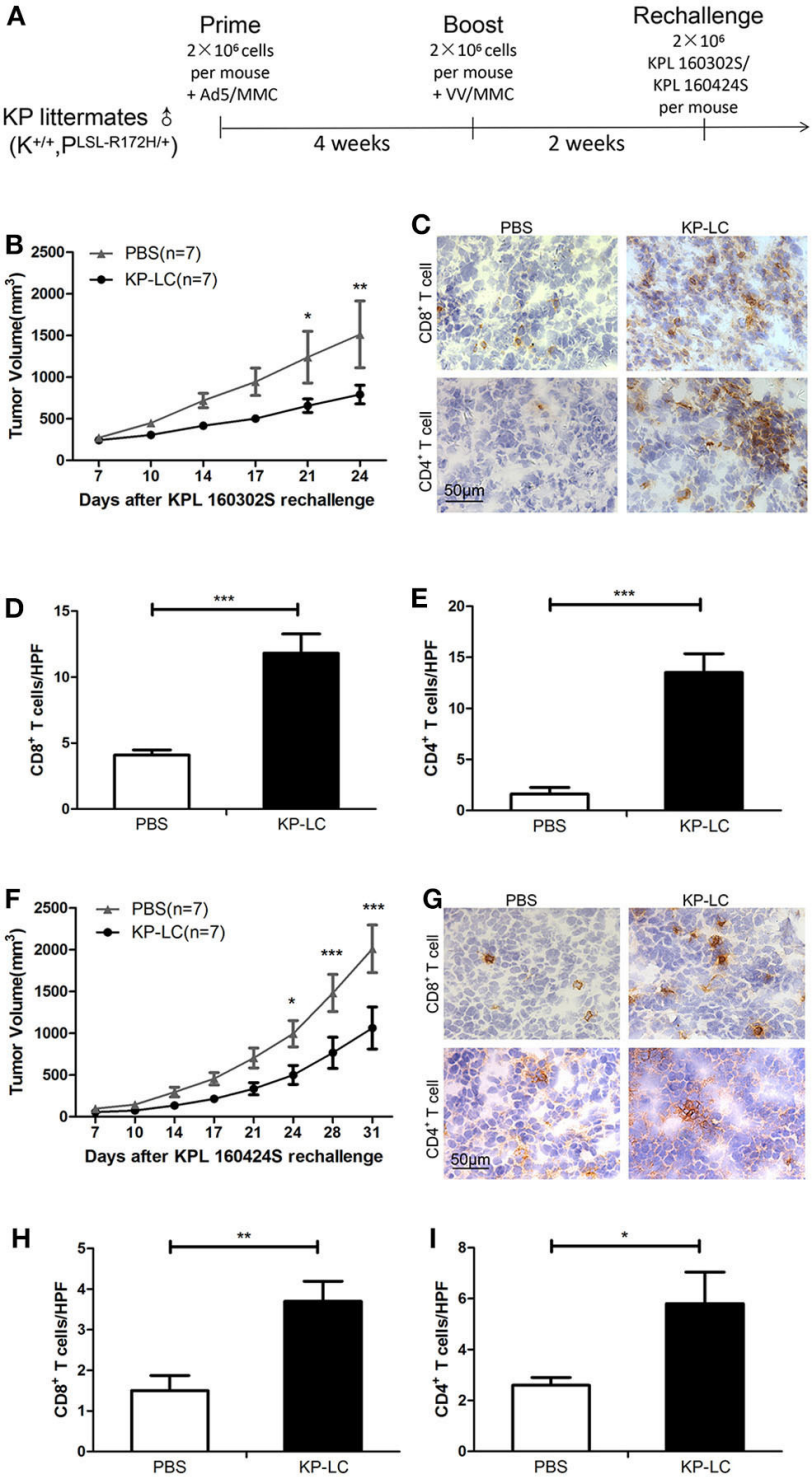
A KP-LC VIReST immunization regime can protect against tumor growth in a subcutaneous lung cancer model. **(A)** KP littermates were immunized with AdV-infected KP-LC cells, followed by a booster immunization using VV-infected KP-LC cells 4 weeks later. Two weeks after boost, mice were challenged using 2 × 10^6^ KPL 160302S or KPL 160424S cells. **(B)** Mice were treated using the regime shown in **(A)** and KPL 160302S tumor growth monitored. Mean subcutaneous tumor sizes ± SEM are shown for each group and compared using a 2-way ANOVA with Bonferroni *post-hoc* testing (*n* = 7/group). **(C)** Representative images of immuno-histochemical staining for CD8+ and CD4+ T cells in subcutaneous tumors derived from **(B)** 24 days post-inoculation. **(D,E)** Lymphocytes were counted in 10 high power fields (HPFs) randomly selected from each group. Mean ± SEM are shown for each group of CD8+ T cells **(D)** or CD4+ T cells **(E)** and compared using an independent *T*-test. **(F)** KP littermates (K^+/+^, P^LSL−R172H/+^) immunized as in **(A)** were re-challenged using KPL 160424S tumor cells (*n* = 7/group). Mean subcutaneous tumor sizes ± SEM are shown for each group and compared using a 2-way ANOVA with Bonferroni *post-hoc* testing. **(G)** Representative images of immuno-histochemical staining for CD8+ and CD4+ T cells in subcutaneous tumors derived from **(F)** 31 days post-inoculation. **(H,I)** Lymphocytes were counted in 10 HPFs randomly selected from each group of CD8+ T cells **(H)** or CD4+ T cells **(I)**. Mean ± SEM are shown for each group and compared using an independent *T*-test. *p < 0.05, **p < 0.01, and ***p < 0.001.

To demonstrate the principle of prophylactic vaccination, the efficacy of a Virus Infected, Reprogrammed Somatic cell-derived Tumor cell (VIReST) vaccination regime was examined using subcutaneous models of lung cancer in immunocompetent mice. As detailed in Figure 3A, KP littermates that are not disposed to cancer development were immunized at day 0 using AdV-infected, MMC-treated KP-LC cells. Four weeks after prime vaccination, VV-infected, MMC-treated KP-LC cells were injected subcutaneously as a boost. Two weeks following this booster vaccination, lung cancer cell lines KPL 160302S (Figure 3B) or KPL 160424S (Figure 3F) were inoculated into the flank of immunized mice to form subcutaneous tumor and tumor growth measured. While prophylaxis did not completely prevent the growth of tumors after challenge, it was able to delay and impair growth after challenge with a large number of tumor cells compared to PBS vaccination. To determine the mechanisms responsible for efficacy, tumors were analyzed using immunohistochemistry (IHC) at day 24 (KPL 160302S) or 31 (KPL 160424S) after re-challenge. The numbers of CD8^+^ and CD4^+^ T cells infiltrating the tumors were analyzed (Figures 3C–E,G–I). In both models, prophylactic vaccination using the KP-LC VIReST regime resulted in a heavier infiltration of both CD8+ and CD4+ T cells, demonstrating that vaccination can induce adaptive immune responses capable of homing to the site of tumor development to impede growth.

### VIReST Using iPSC-Derived Lung Cancer Cells Evokes Anti-tumor Immune Responses in an Induced Transgenic Model of Lung Cancer

**Figure 4:**
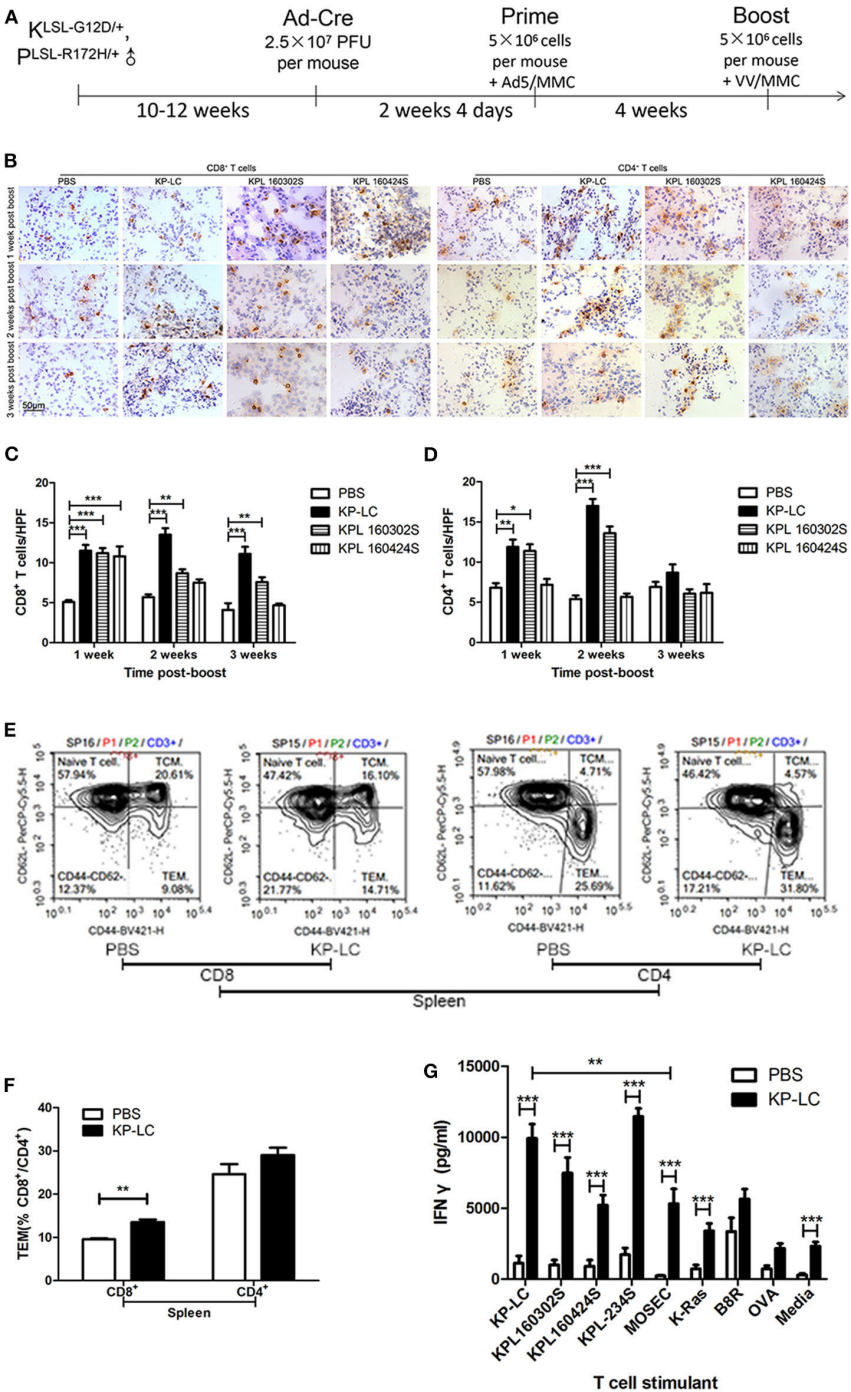
A VIReST regime using induced KP-LC tumor cells induces potent anti-tumor immune responses. **(A)** Schematic representing the immunization protocol. LSL-KRAS^G12D/+^; LSL-Trp53^R172H/+^ mice were infected with Ad-Cre to induce lung cancer development. Two weeks 4 days later, mice were immunized with AdV-infected, mitomycin C-treated KP-LC iPSC-derived tumor cells, KPL 160302S, lung tumor cells, or KPL 160424S lung tumor cells. Four weeks later, a booster of VV-infected, mitomycin C-treated KP-LC, KPL 160302S, KPL 160424S cells were subcutaneously given. Control animals were treated with PBS at the same time points. **(B)** Representative images of immunohistochemical staining for CD8+ or CD4+ T cells in lung tissues from immunized cre-infected KP mice 1, 2, and 3 weeks post-boost. **(C,D)** Lymphocytes were counted in 10 high power fields (HPFs) randomly selected from each group of CD8+ T cells **(C)** or CD4+ T cells **(D)**. Three mice per time point per group were used. Mean ± SEM are shown for each group and compared using a one-way ANOVA with Tukey post-test. **(E)** Spleens of KP-LC immunized mice were collected 3 weeks post-boost and stained for CD3, CD8, CD4, CD44, and CD62L. Representative images of flow cytometry were shown. **(F)** The percentages of effector memory T cells (TEM) (CD44^hi^; CD62L^lo^) of **(E)** are shown (*n* = 3/group). Mean ± SEM are shown for each group and compared using an independent *t*-test. **(G)** Three weeks post-booster vaccination, splenocytes were re-stimulated *ex-vivo* with growth-arrested lung cancer cells as shown, or mouse ovarian surface epithelial cells (MOSEC) or K-RAS or B8R or OVA peptides or media and IFNγ production measured using ELISA. Three mice per group were analyzed in triplicate and the mean IFNγ production ± SEM is shown. Significance was analyzed using an independent *T*-test. *p < 0.05, **p < 0.01, and ***p < 0.001.

We next investigated the KP-LC-based VIReST regime in a more pathologically relevant model of lung cancer using the lung cancer transgenic mouse model previously described (21). KRAS^LSL−G12D/+^; Trp53^LSL−R172H/+^ mice were intra-nasally (i.n.) infected with Ad-Cre to induce KRAS^G12D^ and P53^R172H^ expression within the lung and drive carcinogenesis. H&E staining of lung sections carried out 3–28 weeks after AdCre delivery demonstrated that early lesions could not be detected 3 weeks post-infection (Figure S3) suggesting that disease had not yet developed in these animals or was at an early stage, undetectable by histopathology examination. After 8 weeks, tumors with enlarged nuclei with prominent nucleoli were observed. In contrast, KP mice that were not infected with AdCre did not develop signs of lung carcinogenesis at any age (Figure S3). Based on the observed progression, mice were treated 2 weeks and 4 days post-AdCre infection with a prime-boost VIReST regime as shown in Figure 4A.

One, two, and three weeks following booster injection, CD4+ and CD8+ T cell infiltration into the lungs was observed (Figures 4B–D). CD8+ T cell infiltration into lung tissues was significantly enhanced by VIReST treatment at each timepoint. CD4+ T cell infiltration was also significantly enhanced by treatment, but this advantage was lost 3 weeks post-booster vaccination, although it should be noted that both anti-tumor CD4+ populations and TReg inhibitory CD4+ populations will be detected using this method. Of note, the immune response caused by KP-LC vaccination was similar to that induced by KPL 160302S vaccination using the same regime for they both elicited more CD8+ T cells infiltration 1–3 weeks post-booster vaccination and more CD4+ T cells infiltration 1–2 weeks post-booster vaccination. To further investigate the breadth of anti-tumor immunity, splenocytes from immunized mice were gathered 3 weeks post-boost and the T cell effector phenotype (Figures 4E,F) and *ex vivo* responses to several lung cancer cell lines and specific antigens were determined (Figure 4G). KP-LC VIReST induced more effector CD8+ T cell populations compared to PBS vaccination (Figures 4E,F). In addition, KP-LC VIReST resulted in significantly enhanced IFNγ responses to all lung cancer cell lines and the KRAS epitope (Figure 4G). Of note, IFNγ responses induced by KPL 160424S cells, which was derived from the advanced lung cancer model and had the same driver mutations as KP-LC, was significantly lower compared to other lung cancer cell lines stimulation. KP-LC VIReST also resulted in significantly enhanced IFNγ responses to a mouse ovarian surface epithelial cell line (MOSEC). However, the production of IFNγ induced by MOSEC is significantly lower than KP-LC (Figure 4G). This phenomenon may be caused by some antigenic similarity between MOSEC and mouse lung cancer cell lines and warrants further investigation into shared antigens. Together these results demonstrate the ability of KP-LC VIReST to induce potent anti-tumor immune reactions within the emerging tumor microenvironment (TME) in a physiologically relevant model of lung cancer.

### VIReST Can Delay Tumor Development in an Induced Transgenic Lung Cancer Model in a CD8+ and CD4+ T Cell Dependent Manner

**Figure 5:**
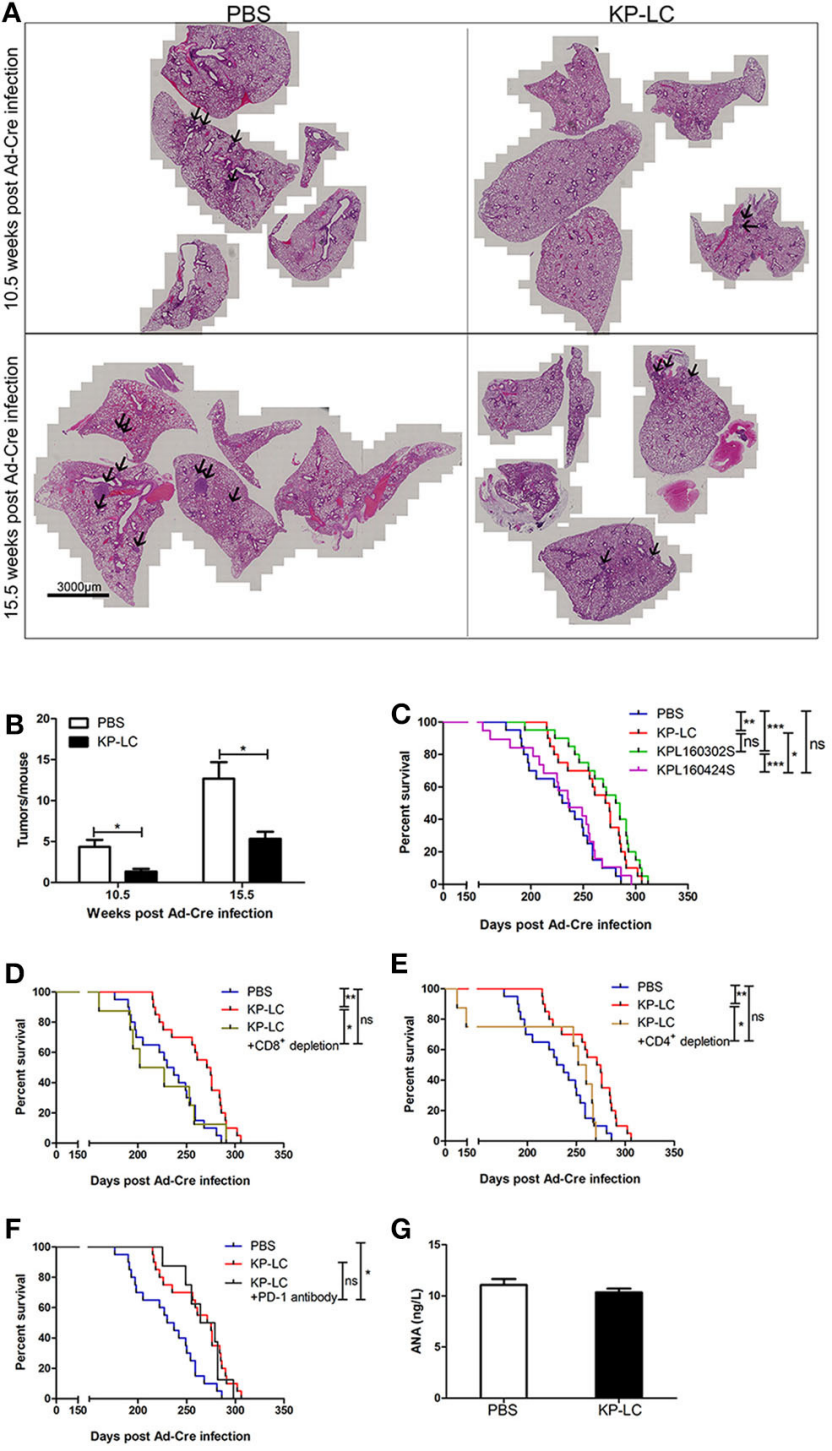
A VIReST regime using iPSC-derived lung tumor cells delays mortality in an induced transgenic model of lung cancer. Ten to twelve week-old KP transgenic mice were immunized using a VIReST regime as shown in Figure 4A. **(A)** Lung tissues of mice 10.5 or 15.5 weeks post-Ad-Cre infection were collected. Representative images of H&E staining are shown. Tumor tissue is indicated by the arrows. **(B)** Tumors present in the H&E stained sections were counted. Significance was analyzed using a student's independent *T*-test. **(C)** After treatment with PBS/KP-LC/KPL 160302S/KPL 160424S vaccination (*n* = 20/group for PBS or KP-LC or KPL 160302S; *n* = 19/group for KPL 160424S), long term survival was monitored. Kaplan-Meier survival analysis followed by Log rank (Mantel-Cox) tests was used to determine significance. **(D)** KP mice were treated with the KP-LC VIReST regime with or without CD8+ T cell depletion and survival monitored as above (*n* = 20/group for PBS or KP-LC; *n* = 8/group for depletion). **(E)** KP mice were treated with the KP-LC VIReST regime with or without CD4+ T cell depletion and survival monitored as above (*n* = 20/group for PBS or KP-LC; *n* = 8/group for depletion). **(F)** KP mice were treated with the KP-LC VIReST regime with or without additional α-PD1 treatment and survival monitored as above (*n* = 20/group for PBS or KP-LC; *n* = 8/group for α-PD1 treatment). Kaplan-Meier survival analysis followed by Log rank (Mantel-Cox) tests was used to determine significance. **(G)** Sera of immunized mice and control mice were analyzed by ELISA for presence of ANAs. *p < 0.05, **p < 0.01, ***p < 0.001, and ns, no significance.

To determine treatment efficacy, animals were immunized following the regime shown in Figure 4A and the progression and survival was monitored. The lung tissues of mice were collected 10.5 and 15.5 weeks after Ad-Cre infection. The tumors of the KP-LC VIReST group were significantly smaller than PBS group (Figures 5A,B). KP-LC VIReST was able to delay disease onset and mortality in this model compared to treatment with PBS, increasing median survival time by 17% (Figure 5C and Figure S4). Furthermore, KPL 160302S vaccination also protected this model, increasing median survival time by 21%, but vaccination using KPL 160424S cells did not have a protective function (Figure 5C and Figure S4). This difference may be attributed to the fact that KP-LC and KPL 160302S had a higher similarity at the transcriptome level (Figure 1F) and elicited similar immune responses (Figures 4B–D). MageE1, a cancer-testis antigen (CTA), was expressed at significantly higher levels in KP-LC compared to KPL 160424S (Figure S2B) and as previously, genes differentially expressed by KP-LC and KPL 160424S were enriched in several pathways associated with cancer (Figure S2D). This may form part of the reason why the efficacy of KP-LC is higher than KPL 160424S. Depletion of both CD8+ (Figure 5D) and CD4+ (Figure 5E) T cells reduced the survival advantage afforded by KP-LC VIReST treatment (Figure S5), demonstrating that the induction of adaptive immune responses is vital for treatment efficacy. Given the sensitivity of some lung tumors to immune checkpoint inhibition (ICI), coupled with an impressive induction of adaptive T cell immunity after vaccination, we reasoned that combination treatment of VIReST with ICI may improve overall mortality. Interestingly however, when induced transgenic mice were treated with α-PD1 antibody, no synergy with VIReST treatment was observed and the efficacy was not enhanced (Figure 5F and Figure S5), although the PD1 blockade did improve the survival of animals compared to untreated control animals. This suggests that treatment failure in this model may not be due to PD1-PDL1-mediated T cell anergy and alternative immunotherapeutic approaches may be considered as synergistic partners.

**Figure 6:**
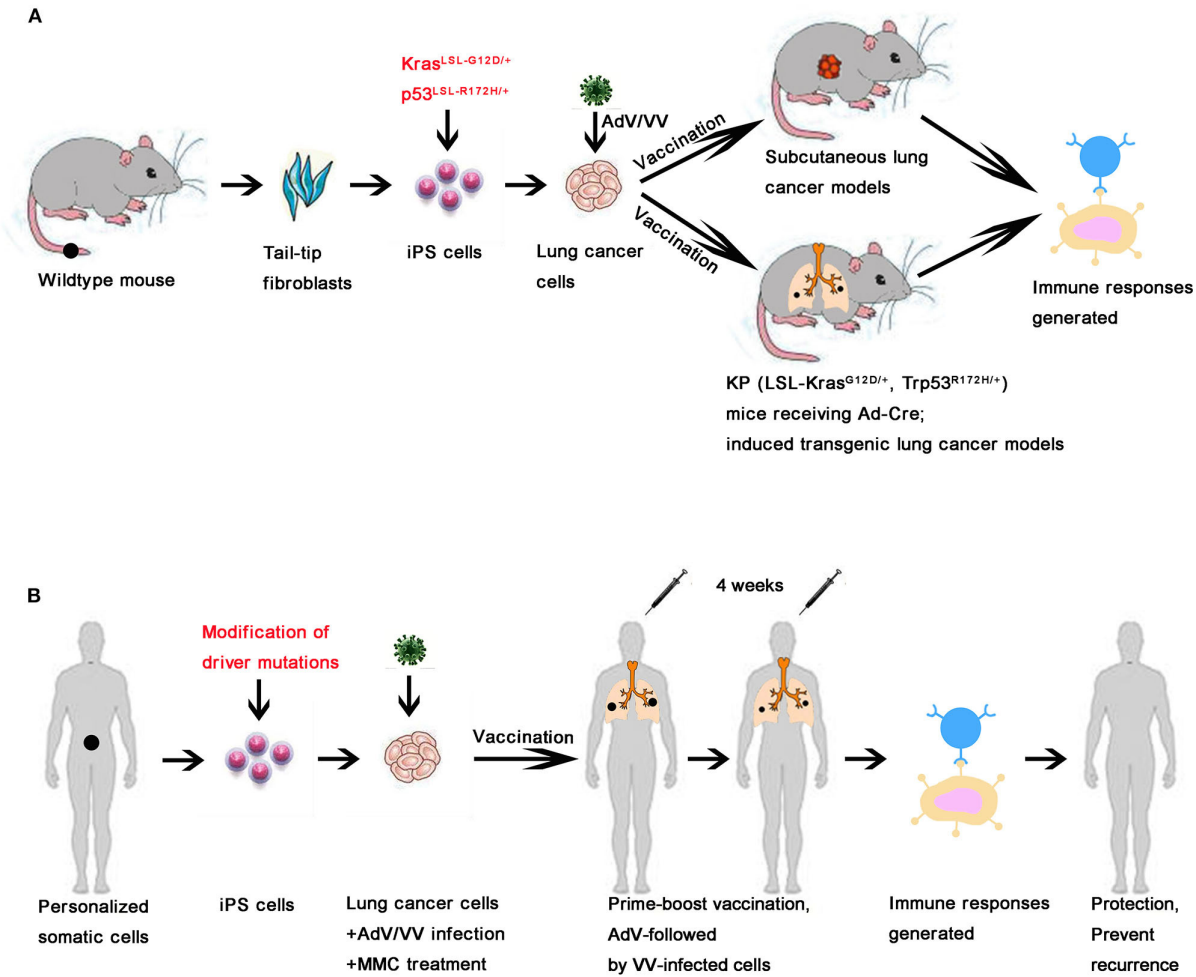
Schematic of the VIReST regime applied for prophylactic prevention or therapeutic treatment of lung cancer. **(A)** A schematic of the VIReST regime and how it has been applied in animal models of lung cancer is presented. **(B)** A schematic of how the proposed VIReST regime would be used clinically is presented.

Of note, an important concern in the development of vaccines derived from ‘‘self''-somatic cells is the possibility of breaking immune tolerance against self-antigens. However, none of the immunized mice developed any evident signs of autoimmune disorders and there was no difference in the amount of circulating anti-nuclear antibodies (ANA) detected between immunized and unimmunized mice (Figure 5G), suggesting that the VIReST regimen was not associated with significant risk of induction of autoimmunity. In addition, no tumor formation was detected at the site of inoculation, demonstrating VIReST as a safe approach for prophylactic prevention of lung cancer as depicted in Figure 6.

## Discussion

Vaccination strategies for lung cancer have historically been unsuccessful, with no positive reports through large, late stage clinical trials over the last decade. However, our improved understanding of the fundamental processes underlying the cancer-immunity cycle has led to a resurgence of interest in developing cancer vaccination strategies, which will be underpinned by rational approaches to selection of tumor antigen targets and prevention of immune suppression. We have recently described a novel vaccination strategy for pancreatic cancer that overcomes current limitations of antigen selection by creating autologous cancer vaccines directly from stem cells (10). Using two common driver mutations, KRAS^G12D^ and P53^R172H^, we were able to replicate the transcriptome profile of cancer cells isolated from transgenic mice in our stem cell derived pancreatic cancer cells. Here, we extend this protocol to lung cancer and importantly demonstrate that despite the use of the same, albeit ubiquitous, driver mutations, the antigenic profile of subsequent tumor cell lines was highly identical to lung cancer cell lines from induced transgenic models, but not related to pancreatic cancer cells derived in the same manner. This demonstrates that the initiating mutations are less relevant to the ensuing accrual of passenger mutations than the epigenetics and lineage specialization of individual tumors. As driver mutations can be expected to have low immunogenicity, these passenger mutations must be key targets if vaccination strategies are to be successful (10, 24). One of the most important aspects of this vaccination regime is the ability to apply entirely autologous vaccines early, or even prior to, disease development. Early application will be the key to vaccine success, as antigen-specific T cell induction can occur prior to selection of MHCI loss of heterogeneity (LOH) variants, common in the evolution of many cancers (25). It has previously been shown that a large proportion of neoantigens were observed to bind to lost MHCI haplotypes (26). Furthermore, recent evidence supports a model of branched evolution in cancers that leads to variable, but potentially considerable intra-tumoral heterogeneity in different tumor types (27–29). This factor may be less relevant for lung cancer vaccination strategies, as recent analysis suggests that 76% of all mutations can be detected in all region of the tumor (9). However, this characterization also revealed that accrual of non-synonymous mutations occur as early events in the evolution of cancer, thus early treatment potentially targets a large number of immunogenic antigens, prior to immune suppression or evasion, setting up an immune system capable of long-term control of disease.

Early disease management is also vital for avoiding the strongly immunosuppressive environment that occurs later in disease evolution. Indeed, early pre-malignant lesions are heavily infiltrated by immune cells with an activated phenotype suggesting an ongoing anti-tumor response. At this stage, immunosuppressive cells are considered rare (7). To maximize the immunogenicity of the vaccine at this stage, we developed a VIReST protocol, in which the stem cell derived cancer cells were pre-infected with either AdV or VV prior to administration in a prime-boost vaccination regime. Virus-infected cell vaccines, whereby tumor cells are pre-infected with replicating tumor tropic virus prior to delivery as a vaccine, has been shown to provoke high levels of anti-tumor immunity that was not achieved when cells were delivered without infection (23), suggesting that replicating viruses can provide relevant danger signals required for vaccine immunogenicity. We have previously demonstrated that replicating viruses can provide adjuvant danger signals and that sequential application of oncolytic AdV followed by VV provides superior efficacy compared to use of either virus alone or in the reverse order when administered as therapeutics in animal tumor models (15) or as vaccine adjuvants (10). We have also previously shown that the VIReST regime is ineffective in a pancreatic cancer model when stem cell-derived cancer cells are delivered without concomitant viral infection (10).

Given that vaccine efficacy will be most prominent in early stage or pre-malignant disease, a patient selection issue arises. Lung cancers symptoms rarely appear until tumors are at an advanced, often incurable stage. However, a number of developments have led to the introduction of screening techniques that can be applied to high risk individuals, including Low-Dose Cat Scans (LCDT) that have been endorsed for annual use to detect early signs of cancer (30, 31). In this regard, use of personalized vaccine as an adjuvant to surgical removal of pre-cancerous lesions can be envisaged. In addition, circulating tumor DNA can be detected in the early-stage lung cancers based on driver mutations (32, 33), and changes in gene expression levels in the bronchial airway can be identified before tumorigenesis (34, 35). These latter advances may also make it possible to more accurately tailor VIReST to the individual, by identification of driver mutations specific to the individual that can be used to derive the lung cancer cells from iPSCs. Patient tailored treatments will be vital for induction of long-term efficacy, considering the role that environmental factors play in the induction of lung tumors through a range of driver mutations.

The results presented here suggest that it is feasible to create personalized cancer cells from iPSCs without the need for specimens from surgical biopsy, and these induced cancer cells can mimic the original tumor, or potential tumor, to a great extent. In addition to its use as an early-stage, or even prophylactic, vaccine we also expect a role for this technology in the detection of drug sensitivity of specific cancers *in vitro*.

To increase the efficiency of manufacture, it is possible to source iPSCs with matched HLA molecules from iPSC stocks (36) and safety concerns are addressed by preventing ongoing cell division using MMC treatment prior to application. No tumor growth was observed at the inoculation site in any experiment conducted and circulating ANA levels suggest no autoimmunity was induced by the treatment.

In this study, we showed that KP-LC vaccine evokes anti-tumor T cell responses using both subcutaneous tumor and induced transgenic models of disease (Figure 6A) and significantly extended the survival time of KP-LC vaccinated mice, although notably did not prevent disease related death. This suggests scope for improvement of the regime, that should include optimizing the number of prime-boost cycles applied to the patient and more specific tailoring of iPSCs by introduction of other driver mutations, particularly those associated with genetic inheritance, including EGFR amongst others (37). It is worth noting that vaccination produced by the lung cancer cell line KP-LC derived from iPS cells or the lung cancer cell line KPL 160302S derived from early lung cancer models can both significantly prolong the survival of lung cancer mice, and their effect on CD8+ T cells and CD4+ T cells infiltration are similar. These two cell lines showed the highest similarities at the transcriptome level and so we inferred that the lung cancer cell line induced by iPS cells can largely mimic lung cancer cell lines from early stage tumors. Interestingly, vaccination produced by the lung cancer cell line KPL 160424S derived from advanced lung cancer models cannot prolong the survival of mice and we speculate that lung cancer cells derived from iPS cells or early staged lung cancer had more immunogenic neo-antigens because they had not been significantly screened or edited by the immune system compared with the lung cancer cells from advanced lung cancer. Interestingly, at the transcriptome level, several genes enriched in cancer associated pathways were expressed more highly in KP-LC than KPL 160424S. Thus, iPS cells can be used as a source of cells for individualized preventive or therapeutic vaccination and for the latter case will be extremely valuable when it is difficult to obtain sufficient tumor cells from early stage cancers to create personalized treatments. Further bioinformatical analysis of the transcriptome data generated from this research may prove invaluable in determining important antigen targets for future vaccination strategies.

Immune checkpoint inhibition (ICI) has emerged as a promising and powerful tool for cancer therapy and some lung cancers have been shown to be sensitive to treatment with ICI therapies clinically. Here we were unable to induce synergy when combining VIReST with α-PD1 antibody therapy, suggesting that ultimate treatment failure in our model was not due to PD1-PD-L1-mediated T cell anergy. Indeed, while immune checkpoint inhibition has been adopted for treatment of non-small cell lung cancer (NSCLC), in fact only a small subset of patients respond to treatment (38). Objective responses have been noted to correlate with higher mutational burdens (39) or as a result of involvement of specific driver mutations that result in induction of PD-L1 expression within the TME (40). Thus, genetic analysis of early stage disease may select patient populations expected to respond more effectively to the combination of VIReST and immune checkpoint blockade. There are however, a number of alternative or complementary avenues for further exploration of combination treatments that may improve regime efficacy, including targeting TIM-3 and Lag-3, additional co-inhibitory receptors known to be clinically significant in lung cancer escape from immune control (41) or inhibition of TReg (42) and MDSC suppressive cells (43) within the developing TME.

In summary, we have described a technological platform for the development of highly antigenically related lung cancer cells from iPSCs of healthy individuals and an effective vaccination regime for prevention and treatment of lung cancer (Figure 6B). These tumor cells were developed via introduction of common tumor driver mutations, but the resulting antigenic profile was not dependent on the driver mutation introduced, suggesting that the epigenetics of an individual, specific to tissue lineage, account for the pattern of neo-antigens expressed. Clearly the situation in human patients is far more complex than reflected in the mouse model used in this study and driving tumorigenesis by introducing further driver mutations, to tailor iPSCs even more accurately, is possible. This technology also has potential for therapeutic use, after tumor resection where adequate viable autologous material is not recovered, as a mechanism to prevent tumor recurrence and metastasis after surgery. Determination of patients for primary prophylactic use is more complex, however identified high-risk individuals would be an obvious population for targeted vaccination strategies. Ultimately, the VIReST provides a safe and effective platform upon which to create robust preventative vaccines that are personalized to be most effective at preventing development or eliminating nascent tumors and preventing their recurrence.

## Data Availability Statement

The datasets presented in this study can be found in online repositories. The names of the repository/repositories and accession number(s) can be found in the article/Supplementary Material.

## Ethics Statement

This animal study was reviewed and approved by the Animal Welfare and Research Ethics Committee of Zhengzhou University.

## Author Contributions

YW conceived and supervised this study. YW and LZ were responsible for all aspects of study design and managed the project. ZheZ and SL designed and conducted most of the experiments and the analysis of results with LD. ZheZ, NW, and WY did *in vitro* and *in vivo* characterization of reprogrammed tumor cells and immune assays. SL supervised the iPS differentiation *in vitro*. ZheZ, ZW, ZhoZ, YaL, JW, YuL, KL, YJ, DS, and PW did animal experiments. ZC and DG performed histopathology studies. YC made oncolytic Adv and VV virus. ZW and YD did the bioinformatics analysis for RNA sequencing. JD and NL participated in interpretation of some experiments and critically reviewed the manuscript. LD, ZheZ, SL, and YW interpreted all results and wrote the manuscript. All authors contributed to the article and approved the submitted version.

## Conflict of Interest

SL, ZheZ, and YW are inventors of a filed patent in the relevant field. The remaining authors declare that the research was conducted in the absence of any commercial or financial relationships that could be construed as a potential conflict of interest.
