# Supplementary material for: Treatment and Prevention of Lung Cancer Using a Virus-Infected Reprogrammed Somatic Cell-Derived Tumor Cell Vaccination (VIReST) Regime
Source: Front Immunol. 2020 Aug 13;11:1996. doi: 10.3389/fimmu.2020.01996 (PMC7438408; doi:10.3389/fimmu.2020.01996)
Supplement: Supplementary file 1 [file Data_Sheet_1.pdf]

Treatment and prevention of lung cancer using a Virus-Infected Reprogrammed Somatic cell-derived Tumor cell vaccination (VIReST) regime

Running Title: Stem cell-based lung cancer vaccination

Zhe Zhang[1], Shuangshuang Lu[2], L S Chard Dunmall[3], Zhizhong Wang[2], Zhenguo Cheng[2], Zhongxian Zhang[2], Wenli Yan[2], Yongchao Chu[2], Dongling Gao[2], Na Wang[2], Yang Li[2], Jiwei Wang[2], Yuenan Li[2], Yupei Ji[2], Danyang Shan[2],Keke Li[2],Panpan Wang[2], Yunshu Dong[4], Jianzeng Dong[5], Nick R Lemoine[2,3], Duanqing Pei[6], Lirong Zhang[7#], and Yaohe Wang[2,3#]

Supplementary Figures and Tables

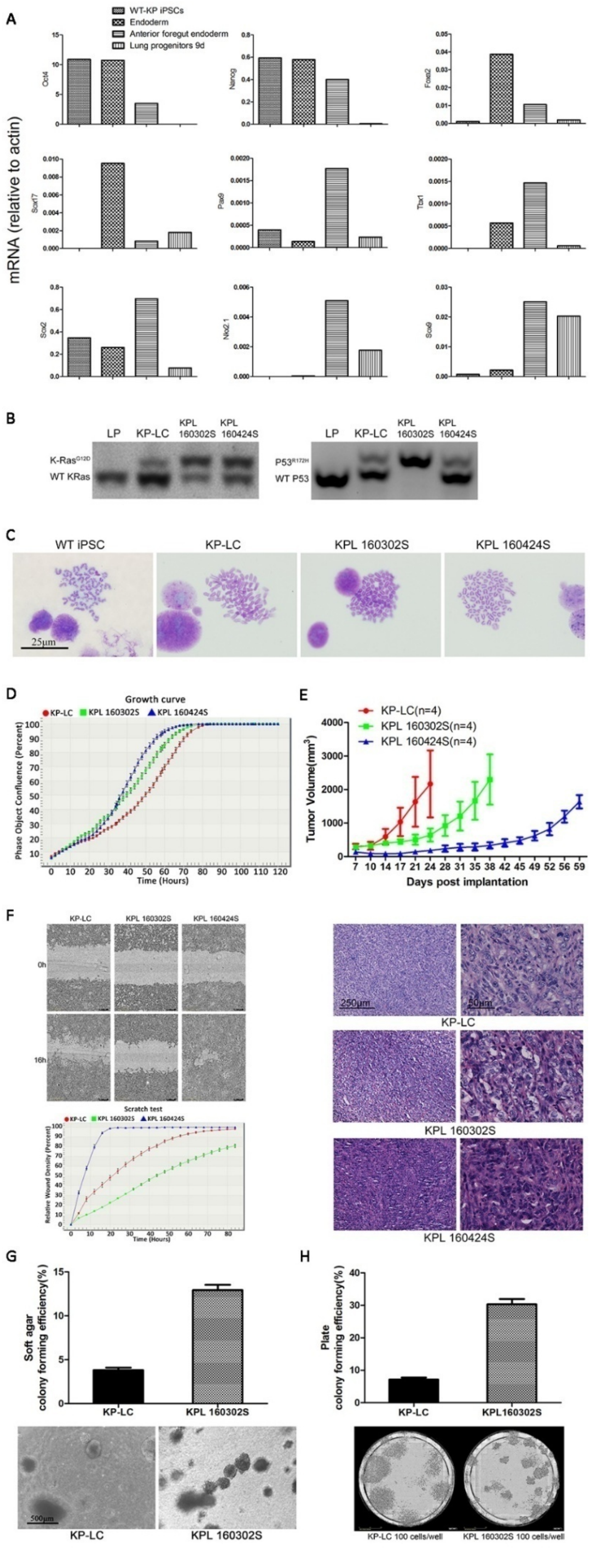
A
WT-KP iPSCs
Endoderm
Anterior foregut endoderm
Lung progenitors 9d
mRNA (relative to actin)
Oct4
Nanog
Foxa2
Sox17
Pax9
Tbx1
Sox2
Nkx2.1
Sox9
B
LP
KP-LC
KPL 160302S
KPL 160424S
K-Ras^{G12D}
WT KRas
P53^{R172H}
WT P53
C
WT iPSC
KP-LC
KPL 160302S
KPL 160424S
25μm
D
Growth curve
KP-LC
KPL 160302S
KPL 160424S
Phase Object Confluence (Percent)
Time (Hours)
E
KP-LC(n=4)
KPL 160302S(n=4)
KPL 160424S(n=4)
Tumor Volume(mm^3)
Days post implantation
F
KP-LC
KPL 160302S
KPL 160424S
0h
16h
Scratch test
Relative Wound Density (Percent)
Time (Hours)
250μm
50μm
KP-LC
KPL 160302S
KPL 160424S
G
Soft agar colony forming efficiency(%)
KP-LC
KPL 160302S
500μm
KP-LC
KPL 160302S
H
Plate colony forming efficiency(%)
KP-LC
KPL160302S
KP-LC 100 cells/well
KPL 160302S 100 cells/well

Figure S1. iPS cells from KP littermate mice can be induced to lung cancer cells. (**A**) Expression of markers for iPSC (Oct4, Nanog, Sox2), endoderm (Foxa2, Sox17), anterior foregut endoderm (Pax9, Tbx1, Sox2, Nkx2.1) and lung progenitors (Nkx2.1, Sox9) were monitored using qPCR. (**B**) Verification of removal of LSL cassette after lung progenitors were infected using Ad5-Cre. KP-LC has mutant bands of Kras and P53 caused by infection of Ad5-Cre activating expression of the mutations. KPL 160302S and KPL 160424S are lung adenocarcinoma cell lines from KP mice (LSL-Kras$^{G12D/+}$; LSL-Trp53$^{R172H/+}$) who have received Ad-Cre intranasally. P53 LOH (loss-of-heterozygosity) occurred in KPL 160302S. (**C**) Karyotype analysis of KP-LC, KPL 160302S and KPL 160424S. WT iPSC was a normal control. KP-LC, KPL 160302S and KPL 160424S all had obvious chromosome number abnormalities. (**D**) Cell growth curve of KP-LC, KPL 160302S and KPL 160424S *in vitro*. (**E**) Subcutaneous tumor growth curve of B6 mice bearing KP-LC, KPL 160302S and KPL 160424S tumors with the initial dose of 2×10^6 cells/ mouse. Mean± SEM is shown. N=7/group. H&E staining of subcutaneous tumors is shown beneath the growth curve. (**F**) Representative wound healing pictures of KP-LC, KPL 160302S and KPL 160424S 0h and 16h after scratch. Relative wound density was analyzed by Incucyte. (**G**) Soft agar colony formation assay of KP-LC and KPL 160302S with the initial density of 2500/5000/10000 cells/well. Colony forming efficiency is shown (n=9). (**H**) Plate colony formation assay of KP-LC and KPL 160302S with the initial density of 50/100/200 cells/well. Colony forming efficiency is shown (n=18).

A

CTAs expression higher in KP-LC than WT-iPSCs

ENSEMBL_GENE_ID	gene_name	logFC	P.Value
ENSMUSG00000031227	Magee1	3.818645005	1.49863E-06
ENSMUSG00000048602	Morc2b	2.332076163	7.41666E-06
ENSMUSG00000031434	Morc4	1.782756529	2.24024E-05
ENSMUSG00000044566	Cage1	1.618408851	4.7076E-05
ENSMUSG00000029279	Brdt	1.406362768	7.63964E-05
ENSMUSG00000017057	Il13ra1	1.918001407	0.000173632

B

CTAs expression higher in KP-LC than KPL 160424S

ENSEMBL_GENE_ID	gene_name	logFC	P.Value
ENSMUSG00000031227	Magee1	2.719467245	4.52193E-05

C

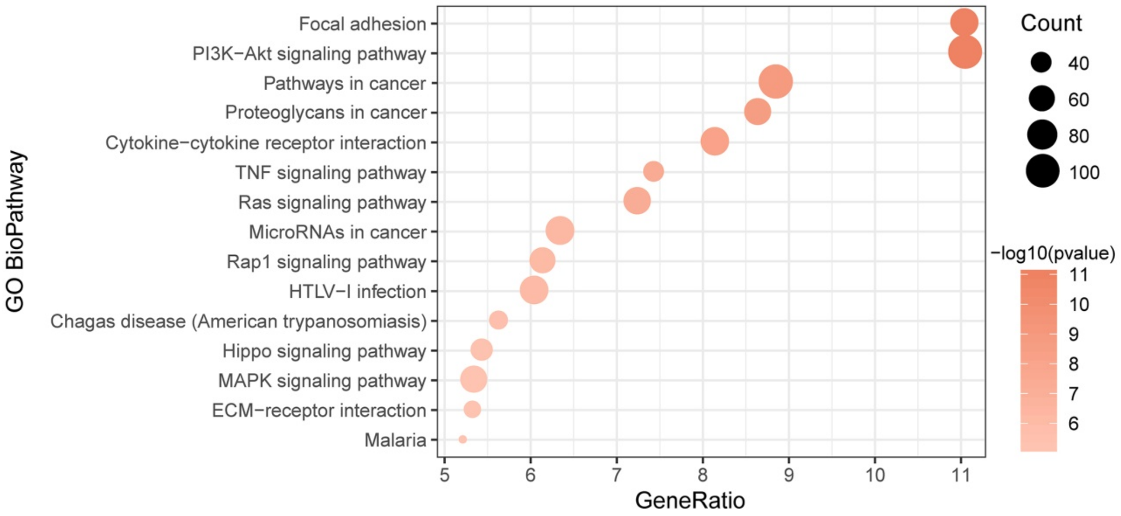

D

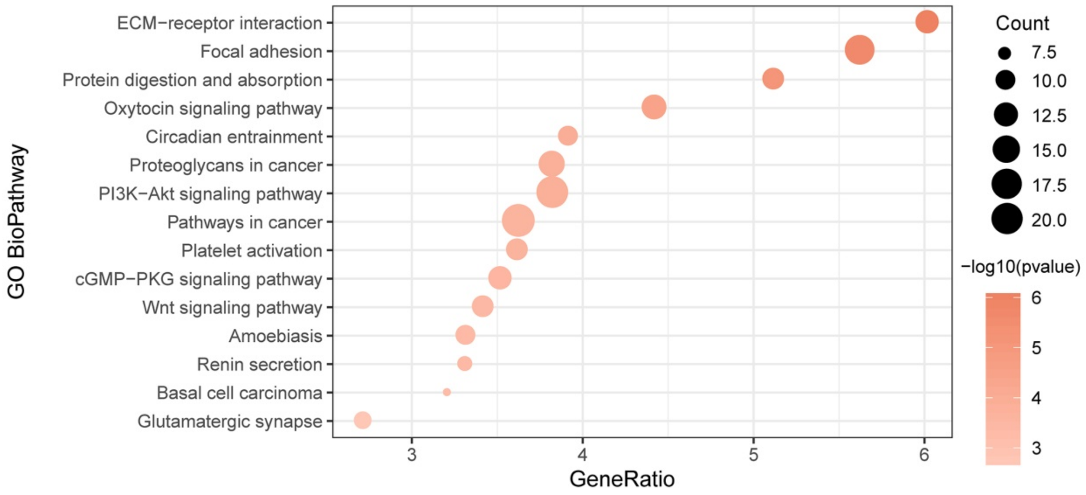

Figure S2. Analysis of gene differential expression. (**A**) Cancer-testis antigens (CTAs) differential expression between KP-LC and WT-iPSCs. (**B**) CTAs differential expression between KP-LC and KPL 160424S. (**C**) Gene Ontology (GO) analysis of gene expression higher in KP-LC than WT-iPSCs (P.Value<0.001). (**D**) GO analysis of gene expression higher in KP-LC than KPL 160424S (P.Value<0.001).

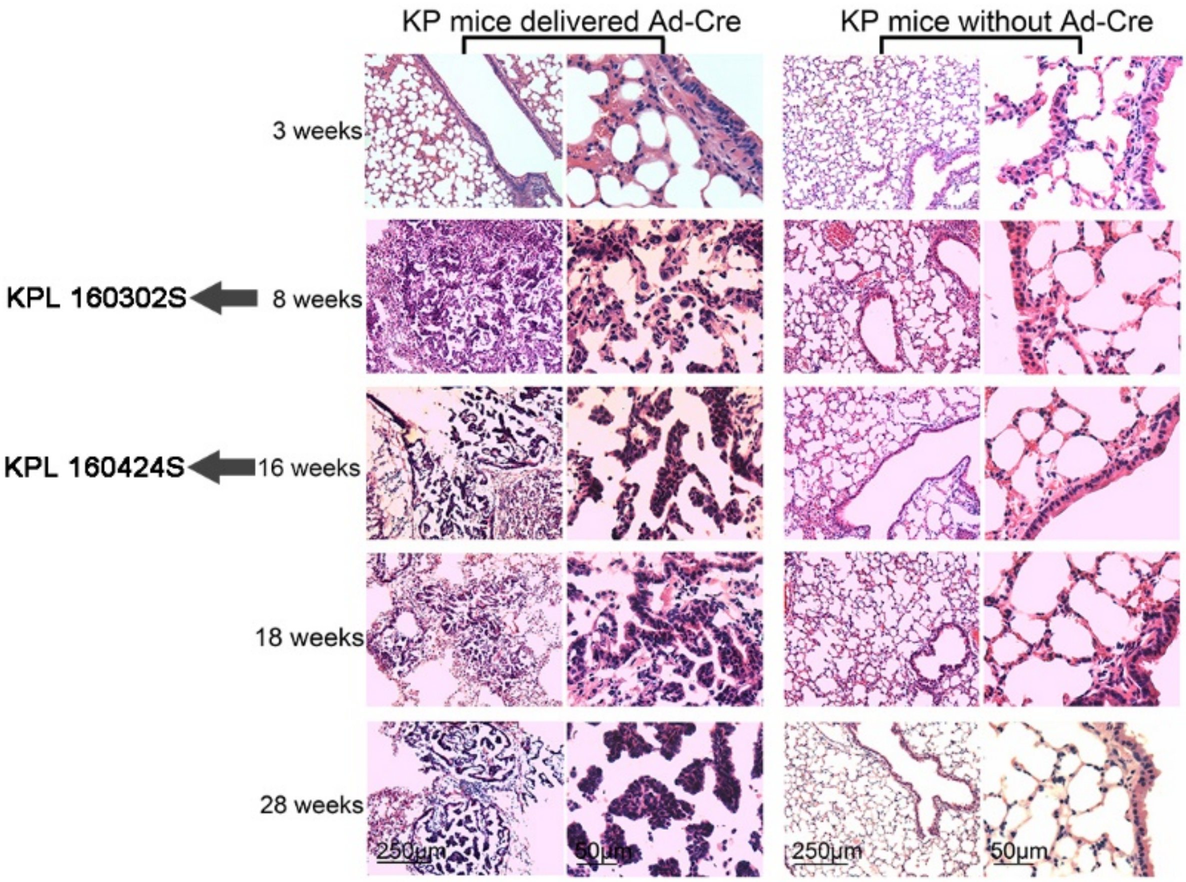

Figure S3. Tumor progression of KP mice after delivery of Ad-Cre. H&E stained lungs from KP mice at various times after intranasal infection with Ad-Cre. KP mice at the same age without delivery of Ad-Cre are shown as a control. KPL 160302S and KPL 160424S were cultured from the 8 weeks and 16 weeks post Ad-Cre inhalation of KP mice.

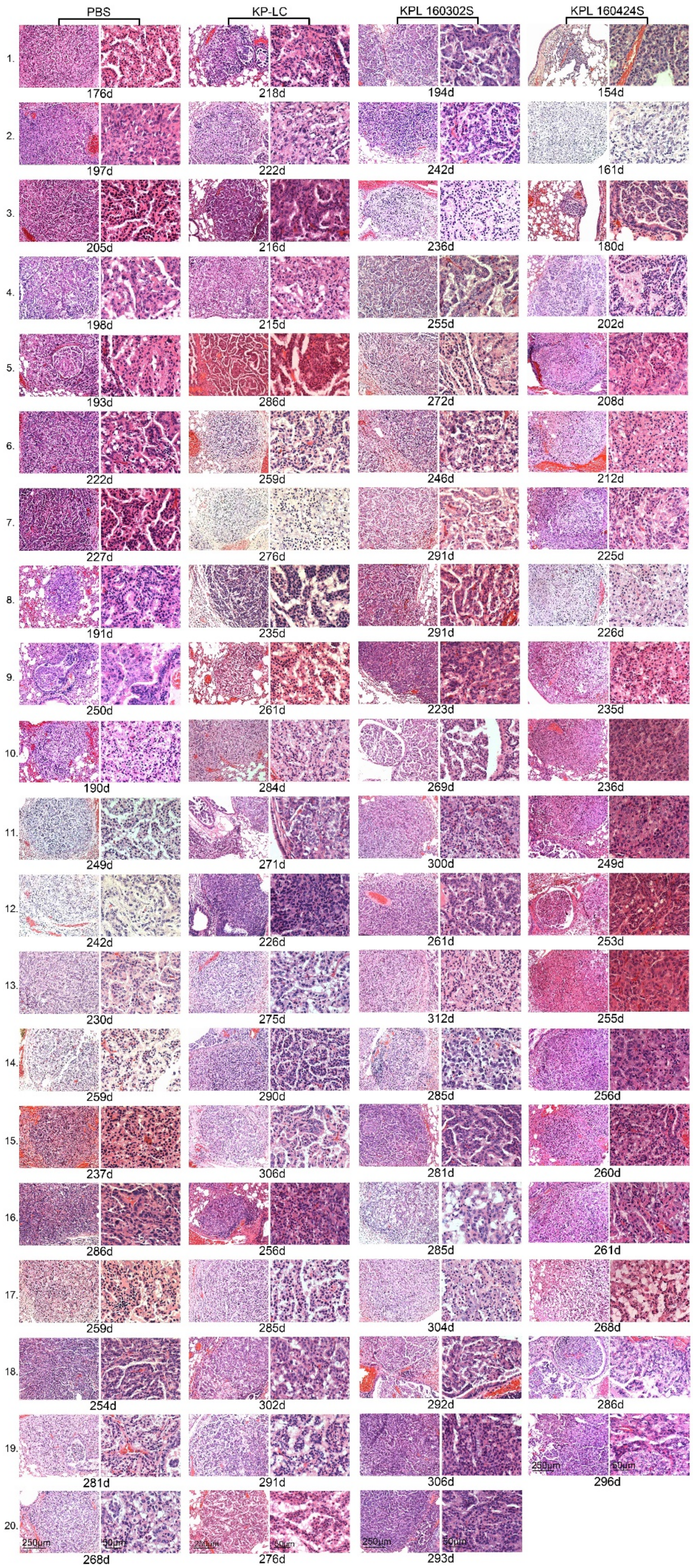
PBS
KP-LC
KPL 160302S
KPL 160424S
1.
176d
218d
194d
154d
2.
197d
222d
242d
161d
3.
205d
216d
236d
180d
4.
198d
215d
255d
202d
5.
193d
286d
272d
208d
6.
222d
259d
246d
212d
7.
227d
276d
291d
225d
8.
191d
235d
291d
226d
9.
250d
261d
223d
235d
10.
190d
284d
269d
236d
11.
249d
271d
300d
249d
12.
242d
226d
261d
253d
13.
230d
275d
312d
255d
14.
259d
290d
285d
256d
15.
237d
306d
281d
260d
16.
286d
256d
285d
261d
17.
259d
285d
304d
268d
18.
254d
302d
292d
286d
19.
281d
291d
306d
296d
20.
268d
276d
293d
250μm
50μm

Figure S4. H&E staining of murine lungs after KP-LC, KPL 160302S and KPL 160424S vaccination treatment. Representative histopathology of H&E staining of lungs in the survival experiment with KP-LC, KPL 160302S and KPL 160424S vaccination.

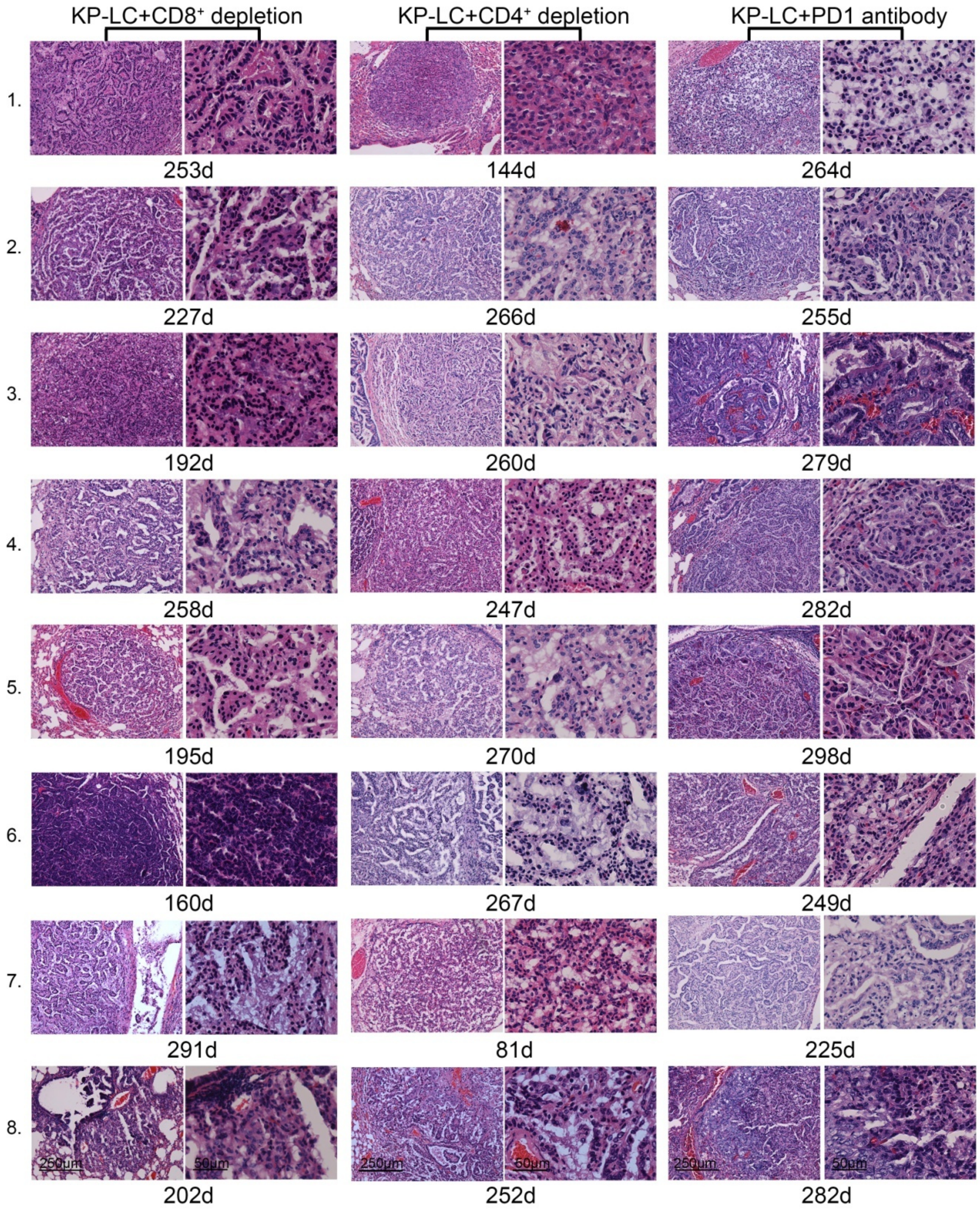

Figure S5. H&E staining of murine lungs after KP-LC VIReST with CD8+ T cells or CD4+ T cell depletion or PD1 antibody. Representative histopathology of H&E staining of lungs in the survival experiment with CD8+ T cells or CD4+ T cells depletion or PD1 antibody.

Table S1. Cell lines created or used in this study

Name	Derivation	Mutations	Notes
**WT iPSC**	129J/C57Bl6 male mice	None	Un-muatated somatic cells driven to pluripotency.
**WT-KP iPSC**	129J/C57Bl6 male mice	Inactivated $Kras^{G12D/+}$ $Trp53^{R172H/+}$	Heterozygous KRas and p53 mutation introduced into WT iPSCs.
**KPC**	LSL-$Kras^{G12D/+}$; $Trp53^{R172H/+}$; Pdx-1-Cre mice	Activated $Kras^{G12D/+}$ $Trp53^{R172H/+}$	Somatic cells containing silent mutations driven to pluripotency. Mutations activated upon differentiation into PPLC to create KPC tumor cells.
**KP-AC**	129J/C57Bl6 male mice	Activated $Kras^{G12D/+}$ $Trp53^{R172H/+}$	Muations in WT-KP cells activated after differentiation into PPLCs by infection with Ad5-Cre.
**KP-LC**	129J/ C57Bl6 male mice	Activated $Kras^{G12D/+}$ $Trp53^{R172H/+}$	WT-KP iPSCs were differentiated into lung progenitor cells, and infected with Ad5-Cre to activate mutations.
**TB11381**	LSL-$Kras^{G12D/+}$; $Trp53^{R172H/+}$; Pdx-1-Cre mice	Activated $Kras^{G12D/+}$ $Trp53^{R172H/+}$	PDAC cell line isolated from tumours arisen in pancreas of LSL-$Kras^{G12D/+}$; $Trp53^{R172H/+}$; Pdx-1-Cre transgenic mouse.
**KPL 160302S**	LSL-$Kras^{G12D/+}$,$Trp53^{R172H/+}$ male mice	Activated $Kras^{G12D/+}$ $Trp53^{R172H/-}$	Lung adenocarcinoma cell line from KP mice 8 weeks after intranasal delivery of Ad-Cre.
**KPL 160424S**	LSL-$Kras^{G12D/+}$,$Trp53^{R172H/+}$ male mice	Activated $Kras^{G12D/+}$ $Trp53^{R172H/+}$	Lung adenocarcinoma cell line from KP mice 16 weeks after intranasal delivery of Ad-Cre.
**KPL-234S**	LSL-$Kras^{G12D/+}$,$Trp53^{R172H}$ male mice	Activated $Kras^{G12D/+}$ $Trp53^{R172H}$	Lung adenocarcinoma cell line from KP mice after intranasal delivery of Ad-Cre.
**MOSEC**	C57Bl6 female mice		By repeated passaging *in vitro*, the late passage mouse ovarian surface epithelial cells can form tumors *in vivo*.

The cell lines that were used or created during this study are listed along with their origins and mutation status. PPLC: pancreatic progenitor-like cells; iPSC: induced pluripotent stem

cell; Ad5-Cre: non-replicating adenovirus armed with Cre recombinase; PDAC: pancreatic ductal adenocarcinoma. LC: lung cancer.

Table S2. qRT-PCR primers used for detection of mRNA in iPSCs and differentiated iPSCs

Gene	Primer F	Primer R
Actin	CCTCATGAAGATCCTGACCGA	TTGCCAATAGTGATGACCTGG
Oct4	TAGGTGAGCCGTCTTTCCAC	GCTTAGCCAGGTTCGAGGAT
Nanog	CTCAAGTCCTGAGGCTGACA	TGAAACCTGTCCTTGAGTGC
Foxa2	TGGTCACTGGGGACAAGGGAA	GCAACAACAGCAATAGAGAAC
Sox17	GAACAGTTGAGGGGCTACAC	GTTTAGGGTTTCTTAGATGC
Pax9	ATCCGCTCCATCACCGACCAAG	CCTTCTCCAATCCATTCACTGCG
Tbx1	CGAGATGATCGTCACCAAGGCA	GTCATCTACGGGCACAAAGTCC
Sox2	CACAACTCGGAGATCAGCAA	CTCCGGGAAGCGTGTACTTA
Nkx2.1	CAGGACACCATGCGGAACAGC	GCCATGTTCTTGCTCACGTCCC
Sox9	GACAAGCGGAGGCCGAA	CCAGCTTGCACGTCGGTT
